# Fusion of wildlife tracking and satellite geomagnetic data for the study of animal migration

**DOI:** 10.1186/s40462-021-00268-4

**Published:** 2021-06-11

**Authors:** Fernando Benitez-Paez, Vanessa da Silva Brum-Bastos, Ciarán D. Beggan, Jed A. Long, Urška Demšar

**Affiliations:** 1 School of Geography and Sustainable Development, Irvine Building, University of St Andrews, North Street, St Andrews, KY16 9AL Scotland, UK; 2 The Alan Turing Institute British Library, England London, UK; 3 British Geological Survey, Research Ave South, Riccarton, Edinburgh, Scotland UK; 4 Department of Geography and Environment, Western University, London, Ontario Canada

**Keywords:** Animal migration, Data fusion, Earth’s magnetic field, GPS tracking, Swarm satellite constellation

## Abstract

**Background:**

Migratory animals use information from the Earth’s magnetic field on their journeys. Geomagnetic navigation has been observed across many taxa, but how animals use geomagnetic information to find their way is still relatively unknown. Most migration studies use a static representation of geomagnetic field and do not consider its temporal variation. However, short-term temporal perturbations may affect how animals respond - to understand this phenomenon, we need to obtain fine resolution accurate geomagnetic measurements at the location and time of the animal. Satellite geomagnetic measurements provide a potential to create such accurate measurements, yet have not been used yet for exploration of animal migration.

**Methods:**

We develop a new tool for data fusion of satellite geomagnetic data (from the European Space Agency’s Swarm constellation) with animal tracking data using a spatio-temporal interpolation approach. We assess accuracy of the fusion through a comparison with calibrated terrestrial measurements from the International Real-time Magnetic Observatory Network (INTERMAGNET). We fit a generalized linear model (GLM) to assess how the absolute error of annotated geomagnetic intensity varies with interpolation parameters and with the local geomagnetic disturbance.

**Results:**

We find that the average absolute error of intensity is − 21.6 nT (95% CI [− 22.26555, − 20.96664]), which is at the lower range of the intensity that animals can sense. The main predictor of error is the level of geomagnetic disturbance, given by the Kp index (indicating the presence of a geomagnetic storm). Since storm level disturbances are rare, this means that our tool is suitable for studies of animal geomagnetic navigation. Caution should be taken with data obtained during geomagnetically disturbed days due to rapid and localised changes of the field which may not be adequately captured.

**Conclusions:**

By using our new tool, ecologists will be able to, for the first time, access accurate real-time satellite geomagnetic data at the location and time of each tracked animal, without having to start new tracking studies with specialised magnetic sensors. This opens a new and exciting possibility for large multi-species studies that will search for general migratory responses to geomagnetic cues. The tool therefore has a potential to uncover new knowledge about geomagnetic navigation and help resolve long-standing debates.

**Supplementary Information:**

The online version contains supplementary material available at 10.1186/s40462-021-00268-4.

## Background

Long-distance migratory navigation consists of two parts, determining the direction of movement (through compass orientation) and geographic positioning, that is, knowing where the animal is located at a specific time [1]. Both these mechanisms support the so-called true navigation, which is defined as finding the way to a far away unknown location using only cues available locally [2]. Compass orientation uses information from the Sun, the stars, the polarised light and the Earth’s magnetic field [3, 4]. Positioning uses geomagnetism [3], olfactory cues [5, 6], and visual cues such as landmarks [7], while another possible cue is natural and anthropogenic infrasound [1], although there are only a few studies on this mechanism. It has been hypothesised that some animals (birds, turtles, and fish) use sensory information from these cues to generate multifactorial internal maps [7–9], although there is an on-going debate about the existence of such maps, as this is very difficult to confirm experimentally [2, 3].

One of the migratory strategies is geomagnetic navigation [3], which uses information from the Earth’s magnetic field for either compass orientation or geographic positioning or both. Various geomagnetic navigation mechanisms have been observed across several taxa [3], from birds [1, 7], fish [9], sea turtles [8, 10], terrestrial [11, 12] and sea mammals [13, 14]. In birds, for example, the strongest evidence for geomagnetic navigation comes from studies that either manipulate animal’s perceived magnetic position and observe their subsequent re-orientation towards migratory destinations [15, 16] or those that surgically manipulate animals’ organs that may help sense magnetic field, such as the trigeminal nerve. One study [17] sections this nerve in Eurasian reed warblers and “virtually displaces” the birds using an artificial magnetic field, then observes that the manipulated birds are not able to correct their direction. Although see a GPS study for the opposite finding in lesser black-backed gulls [18]. In spite of decades of research, we still do not fully understand how exactly animals use the information provided by the Earth’s magnetic field to achieve true navigation [1].

Geomagnetic navigation has been studied with laboratory experiments, which place animals in an artificial magnetic field to study how the magnetic field influences the direction of the onset of migration [2, 7, 15, 17]. Such experiments provide precision and control, but observed behaviour in such experiments may differ from that observed in the wild [1]. A further limitation is that these experiments focus on a small number of individuals from one single species, which limits the generalisability of results across multiple species and taxa [19].

Migration studies now frequently use tracking data, collected with in-situ locational devices (such as GPS loggers) which record the location of animals during their journeys. Tracking, combined with displacement, has become a common way of investigating a particular navigational cue (e.g. see [18] for an example of such an experiment for both geomagnetic and olfactory navigation; some other examples include [20, 21]). Some studies have explored geomagnetic navigation by modelling potential migratory pathways based on real tracking data and a static representation of the geomagnetic field [22]. However, these fail to consider temporal variation in the field, which may be problematic, as solar wind induced short-term variations of the geomagnetic field are greater than the recorded magnetic sensitivity of migratory animals. Neurophysiological experiments have shown that birds can sense changes in geomagnetic intensity between 50 and 200 nanoTesla (nT) [23, 24], and behavioural experiments suggest sensitivity of 15-25 nT [25]. Solar wind disturbances, however, can often reach variations of over 1000 nT in polar latitudes within minutes during geomagnetic storms [26]. That is, the field intensity changes across a very short period (seconds to minutes) for over 1000 nT in the same location (not across a spatial range, but in the same place). Migratory animals may therefore be impacted by such dynamic conditions. For example, looking specifically at birds, if they use the intensity value as a location marker, they may think they are suddenly somewhere else and could try to compensate by changing their flight direction back to their migratory corridor, as shown in virtual magnetic displacement studies [15–17, 27]. If the storm disturbances are strong and come from many directions, this compensation could result in increased variation in attempted flight directions. Alternatively, if they use directional components of the field, such as inclination, they may lose their compass sense and either change direction or switch their navigation to other types of compasses that may be available at that particular location and moment in time, e.g. a Sun or a star compass [4]. Other animals may react in different ways, depending on their particular manner of using the geomagnetic information for navigation [3]. Indeed, geomagnetic storms could be linked to large strandings of whales [13, 14], although this is not fully confirmed - see [28] for a counter argument.

Such effects would most likely be the highest during geomagnetic storms when the temporal variations of the field are the largest. To study how both long- and short-term variation of the geomagnetic field affects migratory navigation, we therefore need to obtain accurate values of the geomagnetic field at the locations and times of animal passage. Satellite geomagnetic data, which provide continuous global coverage, offer great potential for this purpose, but there is currently no tool in existence that would combine these data with animal tracking data.

The process of combining multiple types of data is commonly termed data fusion [29]. In animal migration research, tracking data are frequently combined with dynamic environmental data to account for navigational effects that cannot be understood from tracking alone, such as wind [30] or ocean circulation [31]. Contemporary data fusion in movement ecology is primarily focused on satellite imagery or modelled outputs (wind and atmospheric models). For example, ENV-DATA [32], a popular movement ecology tool, supports the fusion of tracking data with a variety of satellite remote sensing products. Ecologists also explore migration by fusing tracking data with meteorological sources [30, 33]. However, geomagnetic data have to date not been used, perhaps due to their inherent complexity: they are generated as three-dimensional time series of the measurements of the geomagnetic field at a specific location (either a terrestrial station or a satellite). This makes data fusion with similarly structured tracking data (i.e. a time series of observed locations) a geometrical challenge, specifically in terms of bridging the spatial and temporal gaps between respective locations through accurate interpolation.

In this paper we propose a new (and the first) method for spatio-temporal data fusion of wildlife tracking data with geomagnetic data from a satellite source (the European Space Agency (ESA)’s Swarm constellation), which addresses the challenge of the spatio-temporal interpolation between satellite and tracking data. This will provide a possibility to combine satellite geomagnetic data with animal tracking data, something that has never been done before. Our method and its implementation as a free and open source software (FOSS) tool will therefore facilitate new lines of animal navigation research which will be able to explore specific and exact geomagnetic conditions that migratory animals experience during their journeys.

The rest of the paper is structured as follows: we first provide a short overview of relevant concepts, including the Earth’s magnetic field, its measurements and the use of geomagnetic data in migratory navigation research. We then describe our new method and assess its accuracy. We further demonstrate the use of our tool on real bird migration example and conclude with a discussion on how our method could support new data-driven initiatives in research on animal migration.

### A short overview of concepts

**Fig. 1:**
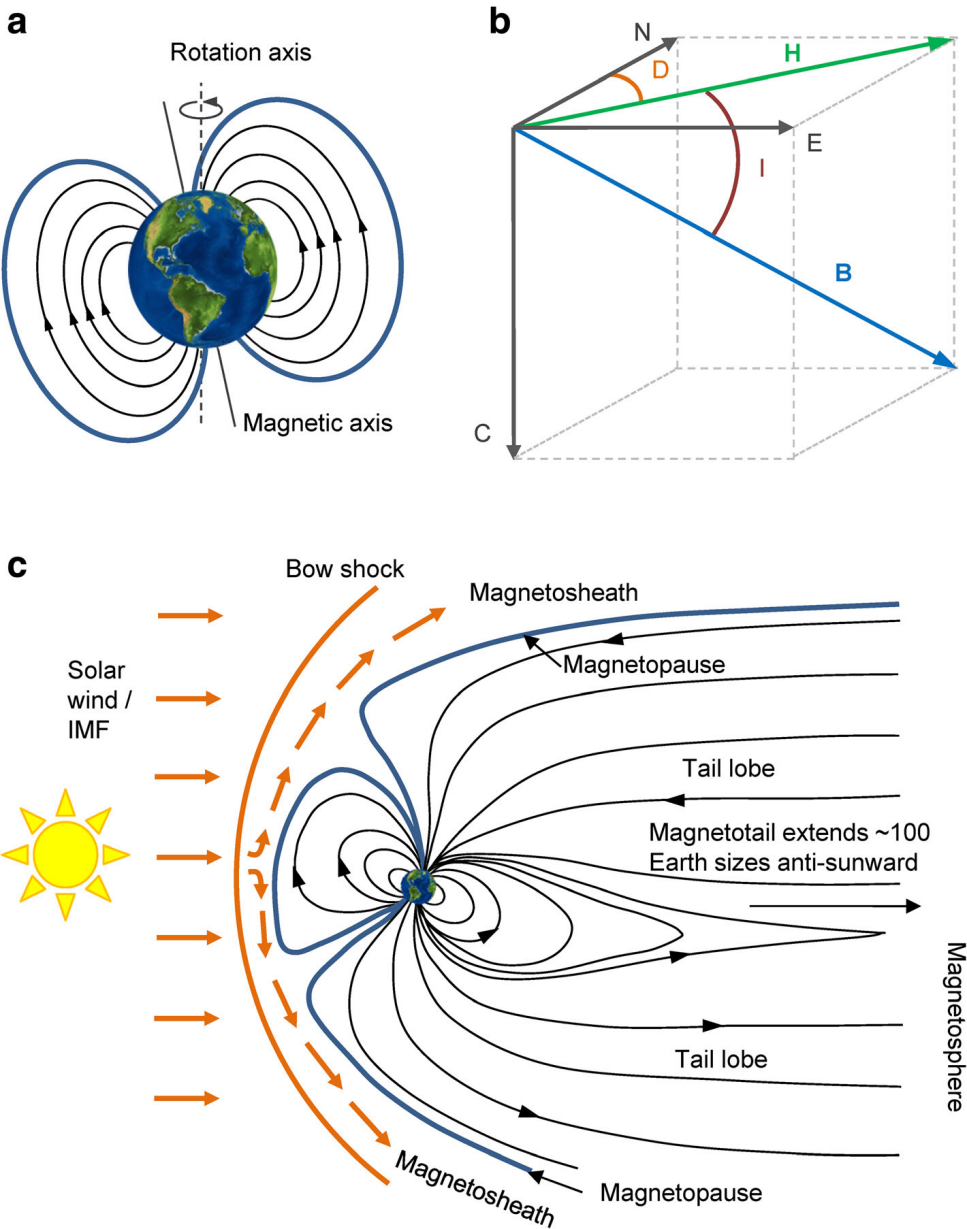
Components of the Earth’s magnetic field. **a** Orientation of the dipole field with respect to Earth’s rotation axis. **b** Measuring the field in the NEC coordinate system. **B** is the field vector, **H** its horizontal component, I the inclination and D the declination. **c** Earth’s magnetosphere is dynamically distorted by the solar wind carrying the Interplanetary Magnetic Field (IMF), which depresses the magnetosphere on the day side and extends its shape on the night side. Magnetosphere is the region of space around the Earth that is affected by its magnetic field. Bow shock marks its outermost boundary, where the speed of solar wind decreases. In magnetosheath, the Earth’s magnetic field is affected by the shocked solar wind and becomes weak and irregular. In magnetopause, the pressure from the Earth’s magnetic field and the solar wind are in balance - the size and the shape of magnetopause therefore constantly change in response to temporal variability in the speed, direction and strength of the solar wind. Magnetotail is the extended anti-sunward part of the magnetosphere: in reality the sphere is not a sphere (as in panel **a**) but has a large extended tail, created through the pressure of the solar wind

Earth’s magnetic field is a constantly fluctuating combination of magnetic fields that originate from different sources: the core field, the lithospheric field and fields generated through external influences [34]. The core field is generated by the geodynamo mechanism of the outer liquid core of the Earth, while the lithospheric field is created by the magnetic properties of the rocks in the Earth’s crust. External fields are caused by interactions with the Sun’s interplanetary magnetic field which produces electric currents in the ionosphere and magnetosphere. On a large scale, the geomagnetic field is approximately dipolar with the magnetic poles offset from the rotation axis (Fig. 1a). However, in detail, it is much more complicated with varying strength and angle across the globe (Fig. 1c). The field is measured in an Earth-based coordinate system, where the magnetic field vector **B** is decomposed along the tri-axial North-East-Centre (NEC) system (Fig. 1b). The length of the field vector **B** is the intensity F. The angle I between **B** and its horizontal component **H** is the inclination and the angle D between **H** and the geographic north (i.e. the N axis) is the declination.

Earth’s magnetic field varies both spatially and temporally. Spatially, the intensity of the core field is between approximately 23,000 nT at the Equator and 62,000 nT at the Magnetic Poles, with geomagnetically quiet time solar-induced variations of about 20 nT at mid latitudes and 100 nT in polar regions [32]. During geomagnetic storms, disturbances range over 1000 nT in polar regions and 250 nT in mid latitudes [23]. These changes can occur over periods of seconds to hours.

Temporal variations of the total field are spread across several components that vary across different time scales. Variations in the core field are called the secular variation and are slow, mostly on time scales longer than a year [34]. The lithospheric field is considered static on periods of less than a millennium. Rapid temporal variations of the field (on timescales of seconds to days) are linked to solar wind, which is a continuous emission of ionised gas from the Sun that fills the interplanetary space in the solar system [26]. Solar wind varies with the activity of the Sun and carries a magnetic field of solar origin, the Interplanetary Magnetic Field (IMF). When this reaches the Earth’s magnetosphere (the area around the Earth that is filled with the geomagnetic field, Fig. 1c), the magnetic field embedded in the solar wind connects with the Earth’s magnetosphere, dragging field lines from day to night side into the magnetotail and driving electrical currents which travel to the surface of the Earth along field lines. When the solar wind is very strong or when the IMF has a particular orientation, the Sun’s magnetic field couples to that of the Earth and creates large disturbances of the geomagnetic field; these are known as geomagnetic storms. During such storms, currents flowing in the magnetosphere and ionosphere intensify, creating auroral displays in high latitude regions, and affect atmospheric density and satellite orbits. They also can interrupt our technology, such as radio communications and GPS signals through ionization. Intense bursts of high energy particles from the Sun associated with Coronal Mass Ejections can also cause similar effects [34].

Real-time local disturbances of the geomagnetic field are represented with a set of geomagnetic indices. The most common of these is the 3-hourly K-index, which quantifies local disturbances in the horizontal component of the magnetic field in the range 0-9, with 0 describing calm conditions and values of 5 or more describing a geomagnetic storm [26]. Values of local K-indices are aggregated into the 3-hour Kp index, which is a proxy for the energy input from the solar wind [35].

Organised scientific measurements of the geomagnetic field started in 1830s, picked up in earnest in the 1930s [36] and have developed into a network of terrestrial magnetic observatories, the International Real-time Magnetic Observatory Network (INTERMAGNET), which currently includes 152 observatories [37]. Over the last 60 years, terrestrial measurements from INTERMAGNET have been complemented with measurements from satellite missions [38] such as POGO (1965-1971), Magsat (1979-80), Ørsted (1999-present), CHAMP (2000-2010), SAC-C (2000-present) and most recently the Swarm mission (launched in 2013) [39]. Terrestrial measurements, such as INTERMAGNET, are advantageous because of high calibration accuracy, but their spatial coverage is irregular (there are very few stations in the oceans and in remote continental regions). In particular, measurements from one INTERMAGNET station are applicable within around 1000 km of a ground station, but the only region where there is a sufficiently dense network of stations to provide full coverage for the whole area is northern and western Europe, which excludes the majority of animal migration pathways. Further, observatories submit their data to the central network at different times and occasionally cease operation – resulting in a temporal lag of several years or occasionally missing data. Satellite missions, and in particular Swarm as a multiple-satellite constellation, resolve this problem as they provide consistent global coverage, available within a few days of measurement.

Satellite and terrestrial geomagnetic measurements are used to generate long-term models of the Earth’s magnetic field [40] capturing the core and large-scale crustal magnetic fields; these models are used in navigation and reference systems. Geomagnetic data are also used to monitor short-term variations in space weather (including occurrence of geomagnetic storms) and to predict potential hazards to the terrestrial and satellite-based technological infrastructure [41]. Satellite and terrestrial geomagnetic data are open and accessible online – this includes both INTERMAGNET and satellite data, as well as K indices [35, 37, 40, 42]. In spite of their openness however, geomagnetic data are rarely used outside the specialist community. This may be because of their inherently complex structure (the Swarm data come as tri-axial time series of magnetic measurements for each of the three satellites in the constellation), a lack of interdisciplinary awareness on what data are available, and/or lack of the technical skills required to obtain and use these data (e.g. they are provided in unfamiliar data formats such as the binary Common Data Format (CDF) developed by NASA [43]).

In the context of migratory navigation, temporally varying geomagnetic data are underused: migration studies typically limit themselves to either a static representation of the field [22] or model its long-term changes: in particular the secular variation [44, 45], where the field changes over decades or centuries. There is therefore a gap that this paper attempts to fill: we develop the first data fusion tool that will allow ecologists studying migration to annotate their animal tracking data with accurate measurements of the Earth’s magnetic field at the location and time of migrating animals. This will, for the first time, support exploration of contemporaneous animal responses to field’s short-term variability.

## Methods

**Fig. 2:**
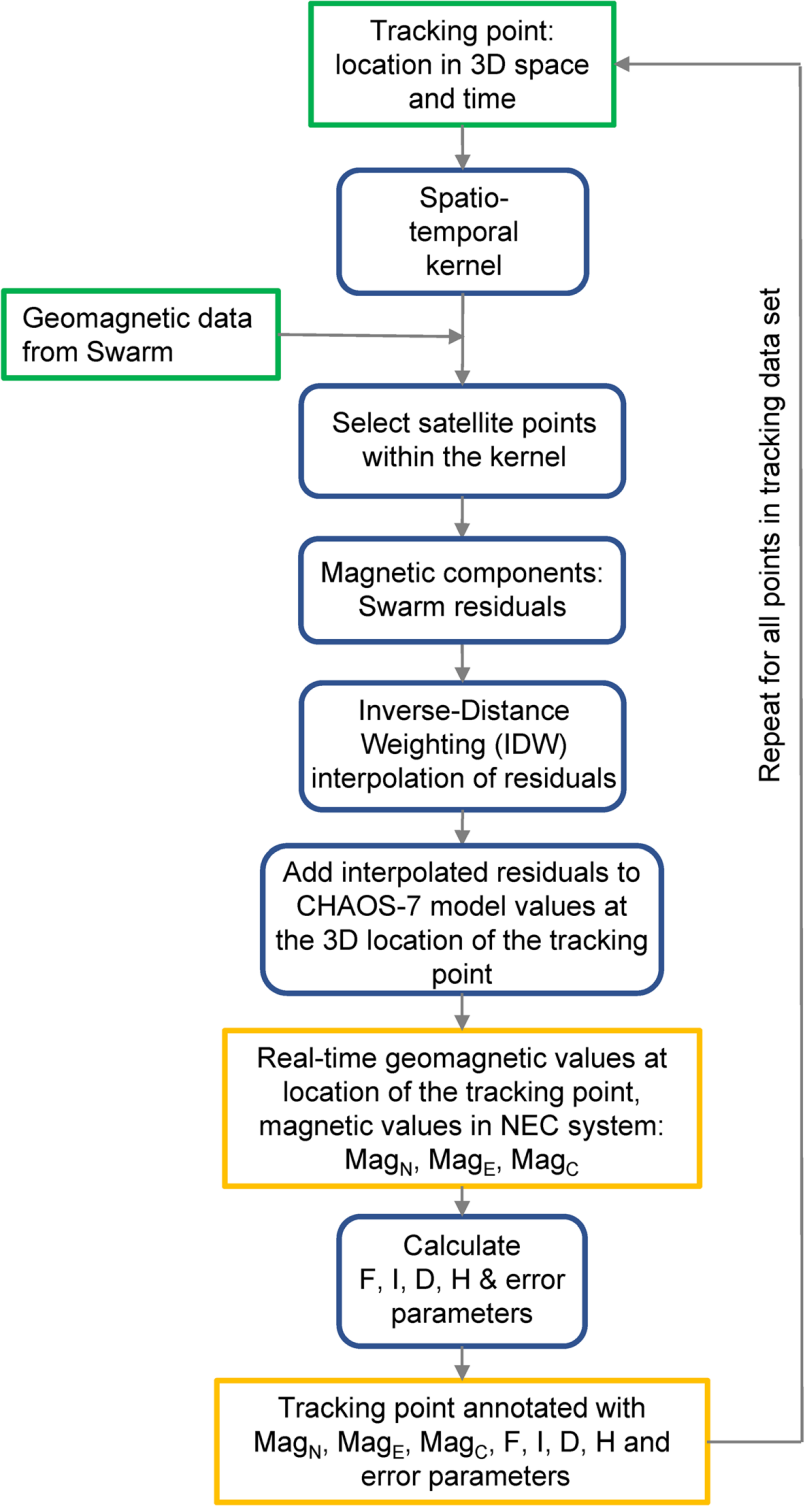
A general outline of our magnetic annotation method. Green boxes show data inputs, blue boxes calculation steps and yellow boxes outputs

Our data fusion method (Fig. 2) combines dynamic geomagnetic data with wildlife tracking data, where each tracked location is annotated with variables describing the estimated state of the Earth’s magnetic field at that location and moment in time. The inputs into the process are tracking data from a tagged animal and geomagnetic data from the Swarm satellite constellation. For each track location, we identify the nearest satellite locations (we call these satellite points) in space and time, i.e. those within a spatio-temporal kernel. We then calculate the spatial distance between the tracked location point and the satellite point as the great circle distance [46] and the temporal distance between the tracked location timepoint and satellite data. The great circle distance is the shortest distance between two points on a sphere, where the path from one to another is located on the surface of the sphere (see Supplementary info 1 for more information).

For each satellite point we obtain residuals between raw measured values of the magnetic field and the modelled values of the field at that location, at the orbital height. These residuals are at the centre of our method, as they represent the actual true variability of the field as influenced by the solar wind, which cannot be modelled but can only be measured in situ. For more information on how we calculate these residuals see the sub-section on Calculation of magnetic components.

Residuals are interpolated using a Spatio-Temporal Inverse-Distance Weighted (ST-IDW) algorithm, which prioritises measurements from satellite points that are the nearest to the tracked location in both space and time. The interpolated residuals are added onto the modelled value of the magnetic field at the location of the tracking point and at altitude of the point (that is, we move the residuals from the orbital altitude to the altitude of the migrating animal). The result is a set of three magnetic values in the NEC coordinate system at the location, altitude, and time of the tracked point, which are then used to calculate other magnetic quantities (intensity F, inclination I, declination D and horizontal component H). In the final step we also calculate error parameters for accuracy assessment. The process is repeated for all points in the tracking data and the result is an annotated track where at each location we now have information on the corresponding geomagnetic conditions.

In the following we explain each of the steps in more detail, while mathematical derivations and technical details are in Supplementary Information 1. We also describe how we assessed the accuracy of our method and present a practical example using real bird migration data.

### Obtaining Swarm data

**Fig. 3:**
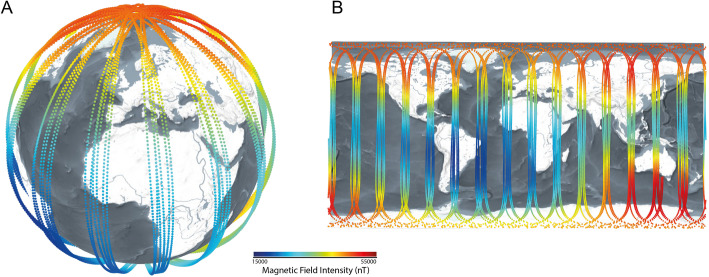
Orbits of the three Swarm satellites over a 24 h period (15 October 2014), **A** shown in 3D and **B** projected on the surface of the Earth. Measurements points are coloured according to the magnetic intensity F. (These images were created with the VirES web client https://vires.services/)

The Swarm mission is a multi-satellite constellation operated by the European Space Agency with a goal to provide near-real-time data on the geomagnetic field and its temporal dynamics [39] (Fig. 3). The constellation consists of three identical satellites: named A(lpha), B(ravo) and C(harlie). A and C move in parallel separated by around 150 km as they cross the equator, flying at a lower orbit of 480 km, while B orbits in a different drifting orbital plane at a height of 510 km and is, at present, counterrotating to the A/C pair. All three satellites are equipped with identical instruments, including the Absolute Scalar Magnetometer (ASM), Vector Field Magnetometer (VFM) and a GPS Receiver. For technical details of the Swarm mission see [39].

Swarm data are open and accessible online following the ESA Earth Observation Policy. We use Level 1b products [47], which are the corrected and calibrated outputs from each of the three satellites, provided in a geocentric NEC reference frame. We use the MAGX_LR_1B product (Magnetic data, low rate), which is a time series of three magnetic measurements in the NEC system from each satellite, collected at 1 Hz resolution by the VFM and calibrated with the ASM and star-trackers. We also use the positional products (GPSXNAV_1B, the on-board GPSR navigational solution) to provide information on the location of all three satellites with 1 Hz resolution. A further product are magnetic values from the CHAOS—7 model and the respective residuals of the Swarm data to this model (see next section for details). We access Swarm data through ESA’s Virtual workspace for Earth observation Scientists (VirES) client [48].

### Calculation of magnetic components

**Fig. 4:**
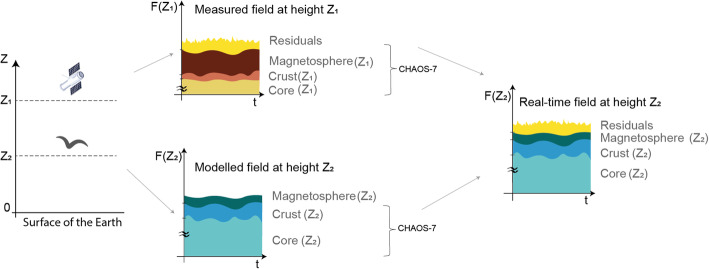
Using Swarm residuals to calculate real-time magnetic field at the altitude of migrating animal. We take the measured field at the satellite height (Z_1_) and subtract modelled contributions of the core, crust and magnetosphere fields at this same height (orange/brown), to obtain the real-time solar-wind induced variability, represented as residuals (yellow). This variability varies at a much higher temporal scale than the modelled contributions and can only be measured in situ. We then obtain modelled field values at the elevation Z_2_ of the migrating animal (values in blue/teal) and add residuals from height Z_1_, which gives us real-time field values at height Z_2_. All modelled values are from the CHAOS-7 model [50, 51]. Charts are not to scale: the contribution of the core field typically represents over 98% of the total field

Swarm data provide information on the magnetic field at the altitude of the orbit, which is above the ionosphere, where the geomagnetic field is affected by the electrical currents induced by the interaction of both sunlight, and the solar wind with Earth’s magnetosphere [34]. This means that to obtain the values of the magnetic field at the Earth’s surface where animals are migrating, the raw measurements from Swarm need to be corrected. We do this by computing residuals between Swarm data and values from a global geomagnetic model, which represents the magnetosphere, core and lithospheric crust fields [49]. That is, we take the measured field at the satellite level and subtract modelled values from the same altitude (Fig. 4, residuals are shown in yellow, while the modelled values of the field at the satellite height are in shades of brown/orange). Thus, Swarm residuals primarily represent the magnetic field variability induced by real-time solar-wind. We then calculate geomagnetic values at the location and altitude of the migrating animal by adding these residuals to the core and crust model values from this location (shown in blue/teal in Fig. 4), thus correcting for the strengthening of the core and lithospheric field nearer their source. Note that we consider the actual altitude of the tracked animal (for example a bird, as in Fig. 4) and do not assume that the animal is on the surface of the Earth, since the intensity of the magnetic field falls off with altitude from the Earth’s surface at around 20nT per km. This is not linear and varies with latitude, but the effect is captured by taking the model at the altitude of the migrating animal (Z_2_ in Fig. 4).

We use the CHAmp, Ørsted and SAC-C (CHAOS-7) time-dependent geomagnetic model in this calculation [50, 51], which is based on observations by Swarm, CryoSat-2, CHAMP, SAC-C and Ørsted satellites, and terrestrial observations from the INTERMAGNET network. Swarm residuals and CHAOS-7 model values are provided through VirES [52].

### Spatio-temporal IDW interpolation

**Fig. 5:**
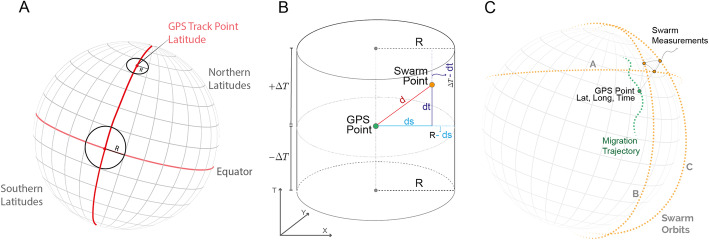
Selection of Swarm points. **A** The spatial extent of the spatio-temporal kernel varies with latitude, with larger circles on the Equator and smaller towards the Poles. **B** Spatio-temporal kernels shown in a space-time cylinder (note that in this display, the third dimension is time), demonstrating the calculation of the spatio-temporal weights (details in Supplementary Information 1). **C** The spatio-temporal kernel allows us to select the nearest Swarm points to the tracking point

Swarm satellites move at around 7.8 km per second along their respective orbits. In order to achieve sufficient interpolation quality at the varied locations of animal tracking data, we must ensure that for each tracking location we include a sufficient number of near-by satellite points (that is, points at which there are satellite measures and which are sufficiently close to the tracking point in space and time) to interpolate the magnetic values. For this, we define a spatio-temporal kernel around the tracking point, within which we search for satellite points. This kernel is defined in two steps (Fig. 5):

I. Time-kernel: we select points from all three Swarm satellites within the +/− 4 h window around the respective tracking point.
II. Space-kernel: from among the temporally-selected points, we select those that fall within a circle centred on the tracking point.

The choice of the parameters for the spatio-temporal kernel was based on the orbital properties of Swarm. The radius of the circle in the space kernel was determined based on the equatorial circumference of the Earth (40,075 km) and the number of orbits of each Swarm satellite in 24 h (each satellite completes 15 orbits in 24 h). This means that the temporal distance between each two consecutive orbits of the same satellite at the Equator is approximately 1.5 h. Given that each orbit has an northward and a southward pass, which are separated by approximately 180^o^, this also means that one orbit counts for two crosses of the Equator, opposite each other. Since the A and C pairs of satellites orbit in parallel with a delay of a few seconds and the orbit of B is currently almost perpendicular, this means that there are four relatively equally spaced intersections of the Equator for each orbit. These intersections are displaced for approximately 667 km with every subsequent orbit (i.e. 40,075 km/(15 × 4)). Based on this, a circle with a radius of 1800 km on the Equator and a temporal window of +/− 4 h from the trajectory point ensures a selection of a minimum of 2-3 satellite points for each location on the surface of the Earth at any time. Since the orbits are polar orbits and therefore converge near the poles, we decrease the circle radius with latitude to 900 km at both Poles (Fig. 5A).

Once satellite points are selected, we obtain respective residuals (see section on Magnetic components above) at each satellite point and interpolate these with spatio-temporal inverse distance-weighting (ST-IDW). That is, we take residual values from all satellite points and interpolate across space and time to obtain residual values at the location of the tracking points. We do this using the following procedure: for every satellite point within the spatio-temporal kernel we compute two measures: i) the spatial distance between the tracking point and the satellite point as a great circle distance (ds; Fig. 5B, the great circle distance is the shortest distance between two points on a sphere, see Supplementary information 1 for mathematical details), and ii) the time distance between the tracking point and the satellite point (dt; Fig. 5B). The spatial and temporal distance are combined into a spatio-temporal distance, which is normalised and inverted to create the weight for interpolation. Interpolated residuals are added to the modelled values at the location, altitude and time of the tracking point to create real geomagnetic measurements in the NEC directions at this location and moment in time (Fig.5C ). We note however that the spatial part of the interpolation is done across a 2D surface, that is, it only considers the horizontal spherical distance between the GPS point and the satellite points and not the difference in altitude. This may introduce a source of error, especially in the part of the residual field originating in ionospheric currents flowing below the satellites but above the elevation of migrating animals.

In the final step, we annotate the tracking point with the resulting NEC magnetic values and other computed magnetic quantities (the intensity F, the horizontal component H, the inclination I and the declination D). For each tracking point we also calculate parameters related to interpolation accuracy – these include the number of Swarm satellite points within the spatio-temporal kernel, the minimum and average spatial distances from the trajectory point to the set of satellite points and the average Kp index at the location and time of the tracking point. The process is repeated for each point in the tracking data.

### Accuracy assessment

**Fig. 6:**
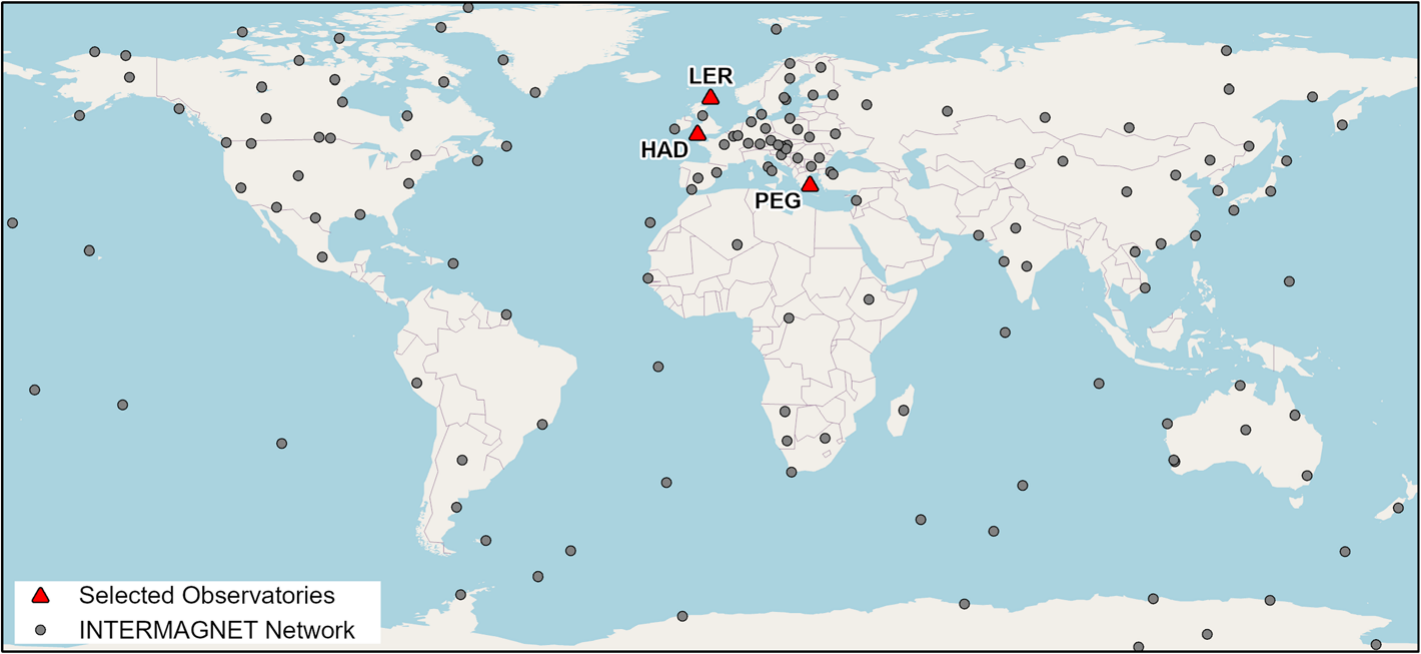
Map of INTERMAGNET observatories showing locations of the three that we selected for accuracy assessment (given with their observatory codes, Lerwick – LER, Hartland – HAR, Pedeli – PEG)

To assess the accuracy of our data fusion procedure, we compare our interpolated geomagnetic values with calibrated magnetic measurements from the INTERMAGNET network (Fig. 6) [37]. INTERMAGNET stations provide data products at different temporal resolutions. We use the quasi-definitive data, which is a time series of absolute values of the magnetic field, provided at the resolution of 1 min [53]. These data have an error of approximately 5nT, depending on the quality control processes at an observatory, but are made available at near real-time. We have chosen quasi-definitive data over the definitive product (the absolute values of the field with full corrections for instrument drift, error < 1nT) as they are available within 72 h of observation, while the definitive product are often not available for a year or more. Given the lower limit of what animals can physiologically sense (20nT [25]), using quasi-definite data is sufficient for our purpose.

We obtained INTERMAGNET data from three observatories and three temporal periods with different levels of geomagnetic activity, which is a common way to assess how magnetic interpolation methods perform under varied conditions [54, 55]. Our goal was to assess how the accuracy of our method varied with: i) the level of disturbance in the geomagnetic field at that location and time, ii) the latitude of the tracking location, and iii) the parameters related to the spatio-temporal kernel (i.e., the number of selected satellite points and the minimum and average spatial distance from the trajectory point to the respective satellite points). The level of activity was defined using the global Kp and local K index (Table 1). We chose INTERMAGNET observatories at three different latitudes (Fig. 6): Lerwick (LER, UK; 60.1° latitude, − 1.2° longitude), Hartland (HAD, UK; 51° latitude, − 4.5° longitude), and Pedeli (PEG, Greece; 38.1° latitude, 23.9° longitude).

**Table 1:** Geomagnetic activity at three INTERMAGNET stations (LER, HAD, PEG) selected for accuracy assessment

Disturbed period					Medium-disturbed period					Quiet period					Interval (hours)
	LER	HAD	PEG	Kp		LER	HAD	PEG	Kp		LER	HAD	PEG	Kp	
22-Jun-2015	0	1	1	2	07-Mar-2015	3	3	3	4	09-Mar-2015	3	3	3	3	0 - 3
	2	3	3	3		3	3	3	4		1	2	1	2	3 -6
	3	4	4	4		1	3	2	3		0	1	1	0	6 -9
	2	3	NA	3		2	3	3	3		0	0	1	0	9 -12
	4	5	4	5		2	3	3	3		0	1	1	1	12 - 15
	5	5	5	6		3	4	4	4		0	1	1	1	15 -18
	7	8	8	8		3	4	4	3		0	0	1	0	18 -21
	6	4	5	6		4	4	4	3		0	1	1	1	21 - 24
23-Jun-2015	8	5	6	7	08-Mar-2015	2	3	2	3	10-Mar-2015	1	1	1	1	0 - 3
	8	5	5	8		1	2	2	2		0	1	1	1	3 -6
	7	5	5	6		2	2	1	2		1	1	1	2	6 -9
	5	4	NA	5		2	3	2	3		1	1	1	1	9 -12
	4	5	4	5		3	3	3	3		0	1	1	0	12 - 15
	2	3	3	3		1	2	2	2		0	1	0	0	15 -18
	2	4	4	4		2	2	2	2		1	1	1	1	18 -21
	3	3	3	4		0	1	0	1		0	0	1	1	21 - 24

Table shows local K indices for each of the three stations in each 3-h slot as well as the planetary global Kp index for the same time period. 3-hourly periods with no data (NA) were excluded from consideration

We obtained one-minute magnetic measurements from all three stations for three 48-h periods, each with a different level of geomagnetic activity (Table 1). We interpolated the Swarm geomagnetic values for each station location across the same time-frame using our method. We then calculated the error as the difference between the interpolated values of intensity F and the INTERMAGNET values (ground truth). We chose to explore the error in intensity only, since calculating errors for each individual axis would require knowledge of the orientation of the sensor on the animal tag, which would need input from additional sensors (such as an accelerometer or a north-seeking gyrometer) and which may not be available in tracking data from tags that only provide GPS measurements.

We used a log-link Gaussian generalized linear model (GLM) to assess how different levels of absolute error of intensity varied with parameters associated with the spatio-temporal kernel and with the local level of geomagnetic disturbance (the K index from each station, Table 1). We further controlled for the observatory, first because the data are collected in different ways at each station and second, because the effects of the geomagnetic disturbance are larger in higher latitudes. We therefore used the station at the highest latitude (LER) as the baseline in the GLM.

### Practical example: is migratory movement of greater white-fronted geese affected by geomagnetic storms?

To illustrate a new type of biological question that can be answered using annotated data from our tool, we explore the hypothesis that migratory movement is affected by local geomagnetic conditions. The novelty of this example is that the conditions that we study are local, that is, they occur at the location and time of each individual - we study this using the local Kp indices from our annotated data. Previous studies used global geomagnetic indices (for example, [56] used global Ap indices, which give a maximum disturbance anywhere on the planet), meaning that there is one value that describes the geomagnetic conditions anywhere on Earth, and there is no way of knowing if those conditions actually occurred at the location and moment in time we are interested in. However, geomagnetic storms are local events, in the sense that the field is disturbed unevenly in both space and time, depending on the fluctuations of the solar wind - a global index aggregates this into one value for the entire planet [42]. In contrast, our tool provides this information locally: by annotating tracking data with real time local geomagnetic information, we can find out where and when each individual encountered stormy conditions. This lets us explore, for the first time, the link between actual local conditions and properties of movement.

We study the North Sea population of the greater white-fronted geese (*Anser albifrons*) [57, 58], which migrate between northern Germany and the Russian Arctic. Our hypothesis is that when geese encounter highly disturbed geomagnetic conditions (as demonstrated by the local Kp index being more or equal to 5, which is the cut-off for a geomagnetic storm [26]), their movement is disturbed and this will be reflected in corresponding movement data. We explore this hypothesis by analysing two movement properties (speed and turning angle) at each location on the geese tracks, during geomagnetic storms and during quiet periods.

We used data from a published study [57, 58] from a single autumn migration (1 Aug 2017 to 15 November 2017) of 22 individuals with a total of 151,156 GPS locations. As white-fronted geese stay in families or pairs the whole year round and migrate in these groups [58], we only used tracks from individuals which did not migrate together, to ensure independence of data. We chose this migration because autumn 2017 was geomagnetically very active, with some of the strongest geomagnetic storms of the 24th solar cycle (2008-2019). A particularly strong storm occurred on 7-8 Sept 2017, with significant effects in the north of Russia [59] – the same area where our geese were situated at approximately the same time.

Geese tracks were first annotated with geomagnetic values using our new tool. Given our data were in WGS1984 long/lat system, all distances and angles were calculated in spherical geometry, as great circle lines (see Supplementary Information 1). We first calculated the speed and the turning angle at each location. Speed was calculated based on the great circle distance between two consecutive GPS points forming a track segment and the duration of the segment (time distance between the two GPS points, given in seconds) and assigned to the first point of each segment. Turning angle was calculated as the angle between two consecutive segments (i.e. using three consecutive GPS points on the track), where the angle was measured on the surface of the sphere between the two segments as great circle distance lines. The angle was assigned to the location that belonged to both segments (i.e. to the middle of the three GPS points). In order to ensure that the potentially unequal duration of the two consecutive segments would not affect the calculation of the turning angles, we also removed all points where segment duration was more than 30 min (1800s). As we were only interested in migratory journeys, we removed non-migratory GPS points in two steps. We first removed points from summer and winter sites by, for each individual, deleting points which were within 200 km of its extreme NE and SW corner. We then further removed all points where geese were stationary, by selecting locations with speed higher than 5 km/h. This is a reasonable choice to isolate data collected during flight, as the highest running speed for geese was found to be 1.17 m/s or 4.21 km/h [60]. Greater white-fronted geese spend the majority of autumn migration in stopover sites [61], so once stationary points were removed, this left us with 13,697 points of migratory movement. For each of these remaining points, we assessed if it was collected during a geomagnetic storm: the points where local Kp was higher or equal to five were marked as occurring during a storm. This resulted in 312 migratory movement points taken during a storm and 13,385 points during quiet geomagnetic conditions. Both storm and non-storm points belonged to all 22 individuals. In the final step we statistically analysed the distribution of speed and turning angle values during the stormy and quiet conditions, to assess if we could identify any differences in these two movement parameters. Specifically, we used standard statistics measures for analysis of segment duration and speed and circular statistics measures for analysis of turning angles.

### Tool and data availability

Our method was implemented using Python 3 in the Jupyter notebooks environment [62]. The code is provided as Supplementary Information 2 and available at GitHub repository MagGeo (https://github.com/MagGeo/MagGeo-Annotation-Program, continuously updated version) or through Zenodo (doi: 10.5281/zenodo.4543735, version from 21 Feb 2021). Our tool uses two specific Python packages: the ESA-VirES Client [63], which connects to the VirES server and handles downloads of Swarm data, and the chaosmagpy package [64], which provides access to the CHAOS model (presently at version 7). We used the *move* R package [65] for movement analysis in the practical example.

Swarm data were obtained through the VirES client [48] and INTERMAGNET data are available at [53]. Data on geomagnetic indices at INTERMAGNET stations were obtained from [35, 66]. Case study data on migration of great white-fronted geese are published on Movebank [57, 58].

## Results

### Accuracy assessment

**Fig. 7:**
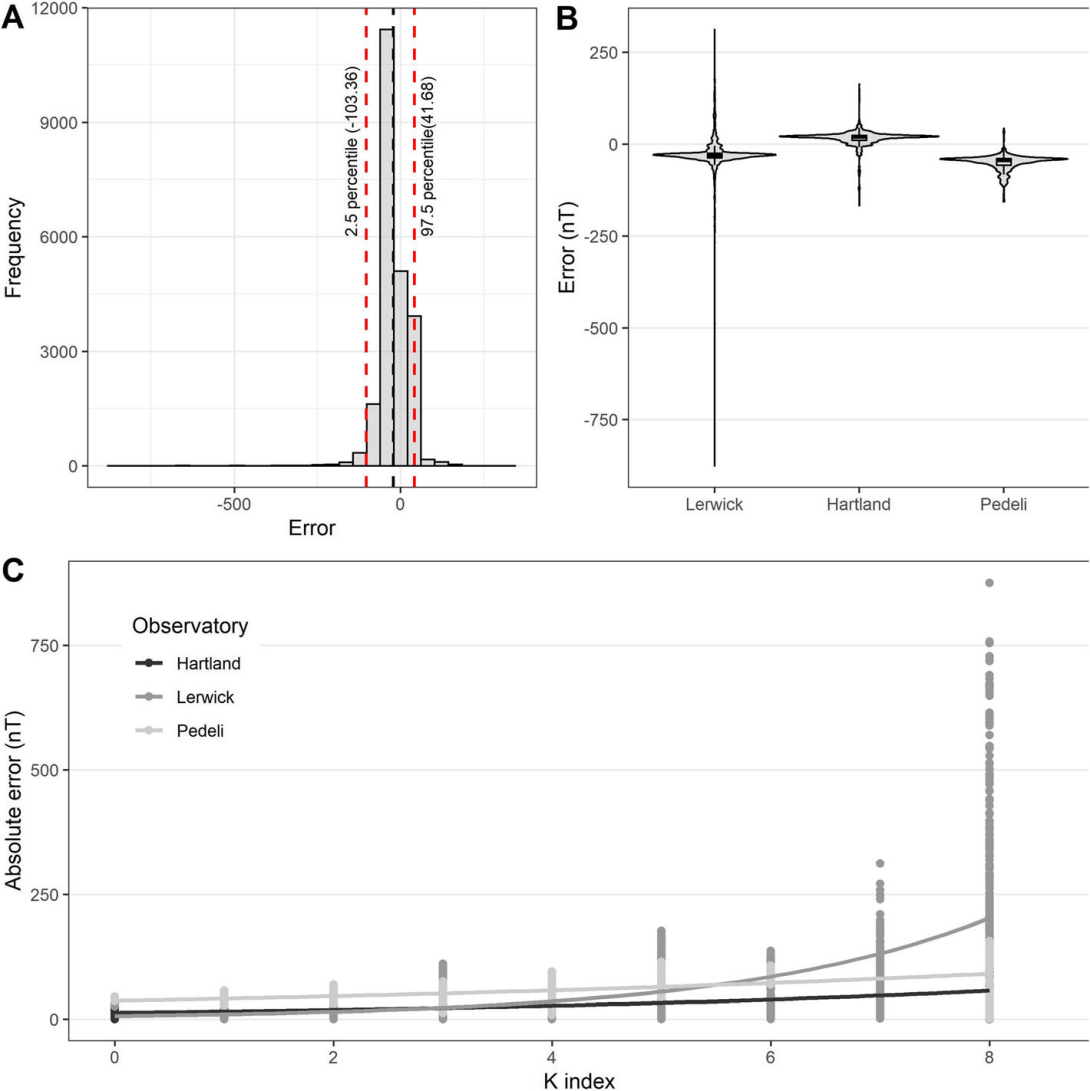
Error in magnetic intensity (F). **A** shows the distribution of the error for all three observatories and the black dashed line indicates the mean error = − 21.61, with a 95% CI [− 22.26555, − 20.96664]. The two red dashed lines show the 2.5 and 97.5 percentile of the distribution. **B** shows the probability density and distribution of error values per station. **C** shows a scatterplot of the absolute error against the K index and the curve of best fit for each observatory (Lerwick, Hartland, Pedeli)

The error in magnetic intensity F (jointly across the three observatories) had a mean of − 21.6 nT (black dashed line in Fig. 7A) with a 95% CI [− 22.26555, − 20.96664]. The distribution of error was concentrated between − 50 nT and + 50 nT (the red dashed lines on Fig. 7A show the 2.5 and 97.5 percentiles), but exhibited negative skewness (γ = − 3.86) owing to a few high negative values (Fig. 7A). The distribution of errors by station (Fig. 7B) shows that the very large and negative errors are at the station at the highest latitude (Lerwick).

On average, we either underestimate or overestimate by a small amount (~ 20-50 nT) at each of the three stations. This small bias away from zero can be ascribed to unmodeled lithospheric field which is not captured by the CHAOS model. We found the error increased with the presence of geomagnetic storms (defined by the local K index; Fig. 7C). Here we found that the absolute error increases logarithmically for Lerwick but linearly at Hartland and Pedeli (Fig. 7C), which is a latitudinal effect related to the proximity of Lerwick to the auroral region.

We explored the relationship between the absolute error of intensity and location and method-related parameters with a log-link Gaussian GLM model. Specifically, our independent variables included the number of satellite points, the minimum and the average spatial distance from the satellite points to the tracking point and the local level of the geomagnetic disturbance given by K index. We also included a control variable for the effect of the IMO observatory, which served as a proxy for latitude (our baseline was LER, which is the observatory in Shetland, at the highest latitude among our three locations). We first removed highly correlated variables (those with *r* > 0.7): specifically, the average spatial distance of all satellite points to the trajectory point was correlated with the minimum distance and the total number of satellite points and was subsequently removed from analysis. The final model (pseudo *R*^2^ = 0.52) included the following variables: the minimum spatial distance to satellite points, the total number of satellite points, the IMO observatory variable, and the local geomagnetic activity index (K).

Both the total number of points (β = − 0.043, *p* < 0.01) and the minimum spatial distance to satellite points (β = − 0.001, *p* < 0.01) were significant predictors of error (Table 2). Each additional satellite point is associated with an absolute error decrease of 4.22%, while each kilometre increment in the minimum distance is associated to a 0.08% absolute error decrease. When controlling for observatories by comparing to the baseline at LER, the regression coefficients for HAD (β =0.804, *p* < 0.01, percent effect = 123.38) and PEG (β = 1.812, *p* = < 0.01, percent effect = 512.29) suggested that the absolute error changes with the observatory. We further found that higher K values (β =0.419, *p* = < 0.01, percent effect = 52.07), which indicate disturbed conditions, were associated with higher levels of absolute error compared to periods with lower K values (during geomagnetically quiet conditions). We also found that higher K values are associated with higher error at higher latitudes (ref = Lerwick:K) than at mid- (Hartland: K, β = − 0.272, *p* < 0.01, percent effect = − 23.81) and lower latitudes (Pedeli: K, β = − 0.320, *p* < 0.01, percent effect = − 27.36).

**Table 2:** Log-link Gaussian GLM model using absolute error as the dependent variable

	β	Std Err	exp(β)	[exp(β)-1]×100
Total Points	−0.043*	0.001	0.95778	−4.22
Station (Ref. Lerwick)				
Hartland	0.804*	0.046	2.2338	123.38
Pedeli	1.812*	0.034	6.1229	512.29
K index	0.419*	0.002	1.5207	52.07
Min. Distance	−0.001*	0.00002	0.9992	−0.08
Interaction (Ref. Lerwick:K)				
Hartland:K	−0.272*	0.0005	0.7618	− 23.81
Pedeli:K	−0.320*	0.035	0.7263	−27.36
Intercept	2.734*	0.035	15.3973	
Observations	19,950			
Log Likelihood	−95,278.82			
Akaike Inf. Crit.	190,573.6			
McFadden’s pseudo R^2^	0.52			

For each predictor we provide the regression coefficient (β), the standard error, exp.(β), and the percent effect size ([exp(β)-1]×100)

*p < 0.01

### Practical example: is migratory movement of greater white-fronted geese affected by geomagnetic storms?

**Fig. 8:**
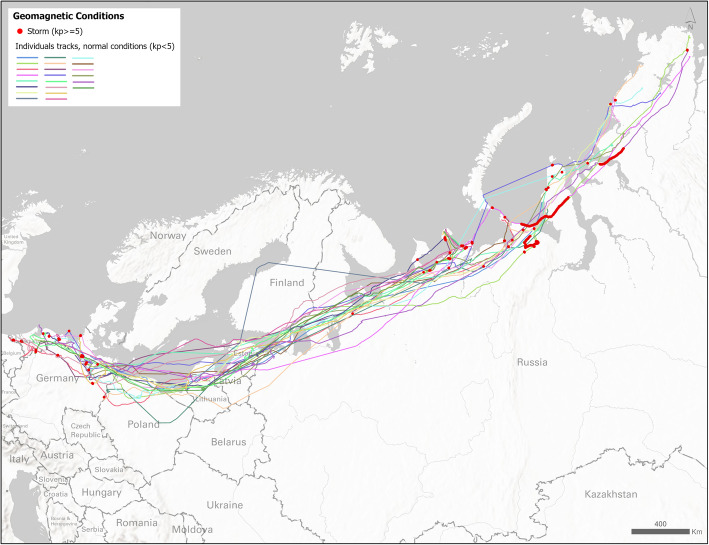
Map showing migration tracks of 22 great white-fronted geese during 2017 autumn migration. Locations where individuals encountered geomagnetic storm conditions (local Kp > =5) during flight are shown in red. Map was created using the Albers equal area projection

Figure 8 shows a map of the tracks that were used in analysis. Locations where individuals encountered geomagnetically stormy conditions during flight are shown in red.

**Fig. 9:**
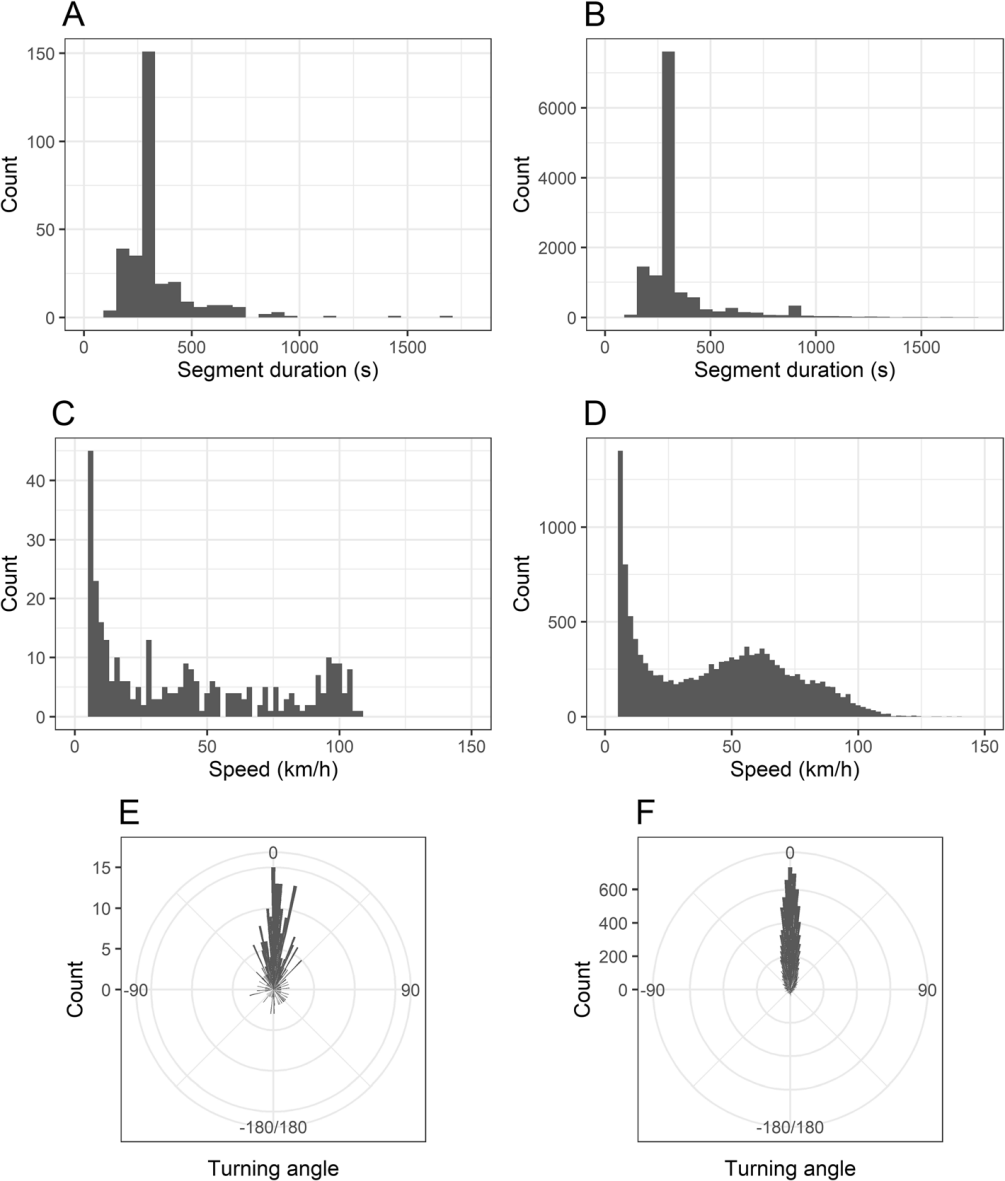
Distribution of movement parameters during and outside of geomagnetic storms. Panels **A** and **B** show the distribution of segment durations for storm (**A**) and no storm (**B**) – the most common duration in both cases is 5 min (300 s). Panels **C** and **D** show distribution of speed during stormy conditions (**C**) and during quiet conditions (**D**). Panels E and F show distributions of turning angle values during stormy (**E**) and quiet conditions (**F**). In these two panels, the 0 reference is the bearing obtained from the previous and current GPS points

Figure 9 shows statistical distributions of the segment duration (time between two consecutive GPS fixes), speed and turning angle values during geomagnetic storms (panels A, C, E) and during quiet conditions (panels B, D, F). Since duration of segments could potentially affect the calculation of turning angle, we first compared the two distributions of respective durations (panels A and B), which are very similar with the mode value of 300 s (5 min) for both distributions. We found that the two means (341.93 s (%95 CI [322.07, 361.79]) and 351.77 s (%95 CI [348.37, 355.16]) for storm/no storm data) and standard deviations (178.28 s and 200.54 s for storm/no storm data) were not significantly different from each other (t-test [67] for means, *p* = 0.16, Levene test [68] for stDev, *p* = 0.30). We therefore assume that since the segment length distributions are similar, they affected the calculation of the turning angle in a similar way for data collected during the geomagnetic storms and those from quiet conditions, which makes turning angles from both cases comparable.

The mean and standard deviation of speed in stormy conditions were 42.27 km/h (%95 CI [38.50, 46.05]) and 33.87 km/h respectively, compared to the mean and standard deviation in quiet conditions: 43.68 km/h (%95 CI [43.20, 44.17]) and 28.82 km/h. The difference between mean speed during quiet and storm periods was not significant (Welch t-test [67], *p* = 0.23), but speed variances were significantly different (Levene test, *p* < 0.001). We used the lawstat R package [68] for Levene test.

The mean turning angle during storms (μ_circ_ = 6.76°, %95 CI [6.67, 6.85]) was significantly different from the mean turning angle during quiet conditions (μ_circ_ = − 0.35°, %95 CI [− 0.34 -0.36]; Watson’s large-sample non-parametric test [69, 70], *p* = 0.003). We also found that the angular standard deviations (a measure of angular concentration) of turning angles for storm (σ_circ_ = 1.03) and quiet conditions (σ_circ_ = 0.92) were significantly different (Wallraff’s non-parametric test [71], *p* = 0.001). Finally, there is further evidence that the two samples of turning angles (quiet vs. storms) were not drawn from a common circular distribution (Watson’s two-sample U2 test [72], *p* < 0.001). All circular tests were performed using the circular package in R [73] and the methods described by Pewsey et al. [74].

These results suggest that, in this data set, movement during geomagnetic storms could have been affected by local disturbed conditions of the magnetic field. Specifically, we may see an effect on the choice of direction at each point, since on average during storms turning angles seem to tend 6.76^o^ towards the right, rather than straight ahead as in non-stormy conditions. Given the general direction of migration from NE to SW, this means that the birds are being displaced towards the north. A further effect is on the choice of turning angles, since these birds tend to choose a wider range of more randomly distributed angles during the storms than in quiet conditions (as shown with a significantly larger standard deviation of angles during stormy conditions). The effect on speed is present in terms of higher standard deviation, but not the mean speed. It is beyond the scope of this paper to speculate on reasons of why we see these effects, based on this small example. However, this small case study demonstrates how geomagnetic effects can now be explored with real time local data and how our tool can support such analyses.

## Discussion

This paper introduced a new method for spatio-temporal data fusion of wildlife tracking data and satellite geomagnetic data. We developed the new fusion methodology, implemented it as a FOSS tool, evaluated its accuracy and demonstrated the use on a case study with real wildlife tracking data.

The practical case study demonstrated how we can address new biological questions about real time responses to geomagnetic conditions, which require linked locational and geomagnetic data through proximity in space and time and which were previously unanswerable. Our case study is the first time that anyone has been able to study how contemporaneous and co-located highly dynamic geomagnetic conditions influence migratory movement of birds. The results of our case study suggest that there may exist an immediate effect of the geomagnetic disturbance on the choice of direction. However, this is a very small example and as such it does not provide sufficient evidence for a more general conclusion. What is important, however, is that this case study demonstrates what is now possible: our tool supports new analyses of animal navigation in response to contemporaneous geomagnetic conditions at the precise location where the animal finds itself, and allows researchers to ask and answer questions that have so far been unanswerable with existing methods and data sets.

In the following we discuss some advantages and limitations that we encountered in the process of developing our data fusion tool, outline recommendations for use and present ideas for future developments.

One of the main advantages of this work is that this is the first time that satellite geomagnetic data have been made accessible to movement ecologists. These data are complex to use due to their structure as a time series of tri-axial measurements taken at satellite locations at orbital altitude - combining these with similarly complex tracking data poses a specific geometric challenge that our new method successfully resolves. In addition, satellite geomagnetic data are less accessible outside geophysics due to the format used for their publication, the Common Data Format (CDF), which was created by NASA in 1985 [43]. This format is binary and not recognisable by data analysis software commonly used in ecology (such as the R environment for statistical computing). Through resolving both these two issues (i.e. geometric complexity and unusual data format), our Python tool therefore provides, for the first time, an opportunity for interdisciplinary secondary use of satellite geomagnetic data and through this opens new possibilities for data-driven investigation of animal migration.

Another advantage of our method is that while it is demonstrated using GPS tracking data, it can be used with any kind of tracking data used in ecology, such as Argos data, data from VHF radio-tracking or from light-loggers. Non-GPS based tracking systems typically have a higher degree of spatial and/or temporal uncertainty, and these uncertainties may impact the definition of the spatio-temporal kernel. For example, Argos data provide estimated error ellipses for each locational fix [75] and this ellipse could be used as the 2D basis of the spatio-temporal kernel instead of the circle as in our method.

Geomagnetic field varies not only by geographic location and time, but also by altitude: contributions of the core and lithospheric fields are stronger at the surface than higher up in the atmosphere. This means that aerial species that migrate at higher altitudes (for example, bar-headed geese fly over the Himalayas at altitudes of over 6000 m [76]) will experience slightly different geomagnetic conditions than those at the sea level. Our tool addresses this by adding Swarm residuals onto the CHAOS-7 model values at the location and altitude of the tracking point. This however requires accurate measurements of altitude in wildlife tracking data, which are often not captured as accurately as horizontal coordinates, or not captured at all – in such cases, the tool defaults to creating values of the geomagnetic field at an altitude of zero metres above the WGS-84 ellipsoid.

The exploratory analysis of the errors produced by our method indicate a small but systematic bias (mean of absolute error = − 21.61 nT) of the geomagnetic intensity which varies by station due to unmodeled contribution from the local lithospheric field. The offset is small compared to the actual values of intensity (e.g. 55000nT at LER) and disturbance variability (e.g. 1000nT at higher latitudes) though could potentially be larger over certain types of geology like volcanic islands. In terms of using our tool for analysis of animal migration, this means that we would be able to identify if migratory animals reacted to lower or higher levels of the intensity, but because of the generally unknown bias we would not be able to specify the absolute level of the local magnetic field to which animals responded. However, this average field error is at the lower level of the minimum intensity that animals can sense (20-200nT, [23–25]). For this reason and since compass and map navigation strategies depend on patterns in geomagnetic values rather than on absolute values (e.g. a constant bearing for the compass), the magnitude of our error offset is unlikely to pose a problem in migration studies.

By fitting the log-link Gaussian GLM to the absolute error we found that the best model representing our error had a moderate R^2^ (0.52). This suggests that there are either other factors which affect the error, such as the limited availability of Swarm data in any given time-space window or the size of the window. Further analysis of the error structure in our interpolation is potentially useful, but would require ground truth data from more than just three INTERMAGNET stations – this has not been possible in for this study as we were only able to obtain data on local K indices for three stations (i.e. only a few stations provide local K indices continuously). We still observe a statistically significant difference between observatories, which is likely linked to differences in latitude and the proximity to the auroral oval whose position can vary rapidly over tens of minutes.

One of the main predictors of the level of error is the K index, which is a measure of the geomagnetic storm activity. Our accuracy assessment suggests that a higher K index results in lower accuracy, especially in higher latitudes. This is reasonable and in agreement with the polar-orbiting design of the Swarm constellation, which provides denser coverage at higher latitudes where geomagnetic intensity and variation happen to be higher. This variation in accuracy with latitude and geomagnetic disturbance should be considered when using these data, especially when the local K index is 5 or higher (Fig. 6C). Nonetheless, between 1995 and 2014, less than 5% of days were geomagnetically disturbed with Kp > 5 [77]. This indicates that for 95% of the days our method would produce highly accurate results. For migratory studies, a recommendation is to be aware of this and exercise caution when using our tool on data on days when geomagnetic storms occur.

Accuracy might be further improved by changing the parameters of the spatio-temporal kernel or using a different interpolation scheme that would better reflect how the geomagnetic field changes across both time and geographic space. In our ST-IDW interpolation we assume an isotropic change of the field. However, there are differences in how the field varies in North-South and East-West directions [55]: for example, ionosphere is persistent over 1000 km in East-West direction and 800 km in the North-South direction. This could be integrated into our method, for example by either changing the form and size of the spatio-temporal kernel (e.g. using an ellipse for its base rather than a circle) or by prioritising weights in a particular direction.

A further issue is the estimation of the ionospheric part of the geomagnetic field, which contributes to its daily variation. This part of the total field is generated by large solar-induced currents in ionosphere (the region of 60-1000 km above the Earth’s surface), which vary with altitude (there are different currents in different regions of the ionosphere), latitude and time of the day (currents are stronger on the day side of the Earth than on the night side) [26]. Due to complex variation of the induced currents, these ionospheric contributions are highly temporally dynamic and difficult to calculate without actually measuring the field. In our case, the ionospheric contribution at satellite altitude is present in Swarm residuals, and we do not correct for this contribution when we transfer weighted residuals to a lower altitude. The reason for this is that modelling the ionospheric contribution on the ground is very complex. While VirES for example provides an empirical model of the associated magnetic field of the ionospheric current system [48], such models do not work well at higher latitudes and not at all during times with high geomagnetic activity. We have therefore opted for a computationally simpler solution, which introduces an additional error to our final values. Given the high temporal variation of the ionospheric contribution however, this error is minimised through our spatio-temporal weighting of residuals, which down-weighs residuals that are far from the GPS point in space and time. Additionally, and as discussed above, while the error due to ionospheric contribution is still present, the accuracy of our method is sufficient in the context for which the tool was built, that is to study animal responses to the geomagnetic field.

A final limitation is demonstrated in the case study, where a proportion of GPS points could not be annotated due to lack of Swarm data. This occasionally occurs due to issues of satellite maintenance, orbital re-calibration, or other engineering reasons. A list of missing data periods is regularly published on the Swarm website [78]. A solution would be to incorporate data from other geomagnetic satellites which are currently in orbit (for example, a Chinese mission called CSES and a Canadian satellite called Cassiope/ePop). This would also potentially result in more satellite points within the spatio-temporal kernels, thus further improving the accuracy.

## Conclusions

To conclude, our new data fusion method of wildlife tracking and satellite geomagnetic data provides ecologists, for the first time, with an opportunity to explore how migratory animals react to specific geomagnetic conditions. This opens a new and exciting possibility for large multi-species data-based approaches that will search for general migratory responses to geomagnetic cues and re-use tracking data that have already been collected, without requiring to start new expensive tracking studies using trackers with magnetic sensors. With the on-going data revolution in bio-logging, based on miniaturisation of devices and new tracking systems dedicated to animal movement research, such large multi-species data experiments are becoming essential to build new knowledge on animal migration. Our data fusion method supports these new studies by providing links to satellite geomagnetic data that would not be accessible to ecologists otherwise and may thereby help resolve some of the big debates about geomagnetic navigation.

## Supplementary Information

**Additional file 1.** Mathematical derivations and technical details.

**Additional file 2.** Code as Jupyter notebook (both sequential and parallel versions).

## Data Availability

Data: Swarm data were obtained through the VirES client [48] and INTERMAGNET data are available at [53]. Data on geomagnetic indices at INTERMAGNET stations were obtained from [35, 66]. Case study data on migration of great white-fronted geese are published on Movebank [57, 58]. Code: Our method was implemented using Python 3 in the Jupyter notebooks environment [62]. The code is provided as Supplementary Information 2 and available at GitHub repository MagGeo (https://github.com/MagGeo/MagGeo-Annotation-Program) or through Zenodo (doi: 10.5281/zenodo.4543735). Our tool uses two specific Python packages: the ESA-VirES Client [63], which connects to the VirES server and handles downloads of Swarm data, and the chaosmagpy package [64], which provides access to the CHAOS model (presently at version 7).

## References

[1] Deutschlander ME, Beason RC (2014). Avian navigation and geographic positioning. J Field Ornithol.

[2] Holland RA (2014). True navigation in birds: from quantum physics to global migration. J Zool.

[3] Mouritsen H (2018). Long-distance navigation and magnetoreception in migratory animals. Nature.

[4] Chernetsov N (2017). Compass systems. J Comp Physiol A.

[5] Gagliardo A (2013). Forty years of olfactory navigation in birds. J Exp Biol.

[6] Bonadonna F, Gagliardo A. Not only pigeons: avian olfactory navigation studied by satellite telemetry. Ethol Ecol Evol. 2021. 10.1080/03949370.2021.1871967.

[7] Wiltschko R, Wiltschko W (2015). Avian navigation: a combination of innate and learned mechanisms. Adv Study Behav.

[8] Lohmann KJ, Lohmann CMF, Putman NF (2007). Magnetic maps in animals: nature’s GPS. J Exp Biol.

[9] Naisbett-Jones LC, Putman NF, Stephenson JF, Ladak S, Young KA (2017). A magnetic map leads juvenile European eels to the Gulf Stream. Curr Biol.

[10] Brothers JR, Lohmann KJ (2018). Evidence that magnetic navigation and geomagnetic imprinting shape spatial genetic variation in sea turtles. Curr Biol.

[11] Burda H, Begall S, Hart V, Malkemper EP, Painter MS, Phillips JB. 7.24 - magnetoreception in mammals. In: Fritzsch B, editor. The senses: a comprehensive reference (second edition): Elsevier; 2020. p. 421–44. 10.1016/B978-0-12-809324-5.24131-X.

[12] Genzel D, Yovel Y, Yartsev MM (2018). Neuroethology of bat navigation. Curr Biol.

[13] Granger J, Walkowicz L, Fitak R, Johnsen S (2020). Gray whales strand more often on days with increased levels of atmospheric radio-frequency noise. Curr Biol.

[14] Vanselow H, Jacobsen S, Hall C, Garthe S (2017). Solar storms may trigger sperm whale strandings: explanation approaches for multiple strandings in the North Sea in 2016. Int J Astrobiol.

[15] Kishkinev D, Chernetsov N, Pakhomov A, Heyers D, Mouritsen H (2015). Eurasian reed warblers compensate for virtual magnetic displacement. Curr Biol.

[16] Kishkinev D, Packmor F, Zeichmeister T, Winkler H-C, Chernetsov N, Mourisen H, Holland RA (2021). Navigation by extrapolation of geomagnetic cues in a migratory songbird. Curr Biol.

[17] Pakhomov A, Anashina A, Heyers D, Kobylkov D, Mourtisen D, Chernetsov N (2018). Magnetic map navigation in a migratory songbird requires trigeminal input. Nat Sci Rep.

[18] Wikelski M, Arriero E, Gagliardo A, Holland RA, Huttunen MJ, Juvaste R, Mueller I, Tertitski G, Thorup K, Wild M (2015). True navigation in migrating gulls requires intact olfactory nerves. Nat Sci Rep.

[19] Bowlin MS, Bisson I-A, Shamoun-Baranes J, Reichard JD, Sapir N, Marra PP, Kunz TH, Wilcove DS, Hedenström A, Guglielmo CG (2010). Grand challenges in migration biology. Integr Comp Biol.

[20] Willemoes M, Blas J, Wikelski M, Thorup K (2015). Flexible navigation response in common cuckoos Cuculus canorus displaced experimentally during migration. Sci Rep.

[21] Kishkinev D, Heyers D, Woodworth BK, Mitchell GW, Hobson KA, Norris DR (2016). Experienced migratory songbirds do not display goal-ward orientation after release following a cross-continental displacement: an automated telemetry study. Sci Rep.

[22] Åkesson S, Bianco G (2017). Route simulations, compass mechanisms and long‑distance migration flights in birds. J Comp Physiol A.

[23] Beason RC, Semm P (1987). Magnetic responses of the trigeminal nerve system of the bobolink (Dolichonyx oryzivorus). Neurosci Lett.

[24] Semm P, Beason RC (1990). Responses to small magnetic variations by the trigeminal system of the bobolink. Brain Res Bull.

[25] Gould JL, Kirschvink JL, Jones DA, McFadden BJ (1985). Are animal maps magnetic?. Magnetite biominealization and magnetoreception in organisms. Chapter 12, 257-268.

[26] Campbell WH (2003). Introduction to geomagnetic fields.

[27] Henshaw I, Fransson T, Jakobsson S, Kullberg C (2010). Geomagnetic field affects spring migratory direction in a long distance migrant. Behav Ecol Sociobiol.

[28] Pulkkinen A, Moore K, Zellar R, Uritskaya O, Karaköylü EM, Uritsky V, Reeb D (2020). Statistical analysis of the possible association between geomagnetic storms and cetacean mass strandings. JGR Biogeosci.

[29] Zheng Y (2015). Methodologies for cross-domain data fusion: an overview. IEEE Trans Big Data.

[30] Vansteelant WMG, Shamoun-Baranes J, van Manen W, van Diermen J, Bouten W (2017). Seasonal detours by soaring migrants shaped by wind regimes along the East Atlantic Flyway. J Anim Ecol.

[31] Briscoe DK, Parker DM, Balazs GH, Kurita M, Saito T, Okamoto H, Rice M, Polovina JJ, Crowder LB (2016). Active dispersal in loggerhead sea turtles (Caretta caretta) during the ‘lost years’. Proc R Soc B.

[32] Dodge S, Bohrer G, Weinzierl R, Davidson SC, Kays R, Douglas D, Cruz S, Jan J, Brandes D, Wikelski M (2013). The environmental-data automated track annotation (Env-DATA) system: linking animal tracks with environmental data. Mov Ecol.

[33] Oloo F, Safi K, Aryal J (2018). Predicting migratory corridors of white storks, ciconia ciconia, to enhance sustainable wind energy planning: a data-driven agent-based model. Sustainability.

[34] Lanza R, Meloni A (2006). The Earth’s magnetism.

[35] German Research Centre for Geosciences (GFZ) Potsdam (2021). Indices of global geomagnetic activity.

[36] Matzka J, Chulliat A, Mandea M, Finlay CC, Qamili E (2010). Geomagnetic observations for main field studies: from ground to space. Space Sci Rev.

[37] INTERMAGNET. International Real-time Magnetic Observatory Network, 2021. https://www.intermagnet.org/ and https://intermagnet.github.io/. Accessed 16 Feb 2021.

[38] Olsen N, Hulot G, Sabaka TJ (2010). Measuring the Earth’s magnetic field from space: concepts of past, present and future missions. Space Sci Rev.

[39] European Space Agency (ESA) (2021). Swarm.

[40] British Geological Survey (BGS) (2021). World magnetic model (WMM).

[41] Riley P, Baker D, Liu YD, Verronen P, Singer H, Güdel M (2018). Extreme space weather events: From cradle to grave. Space Sci Rev.

[42] National Oceanic and Atmospheric Administration (NOAA) (2021). Geomagnetic Kp and Ap Indices.

[43] Space Physics Data Facility (2021). What is common data format?.

[44] Wynn J, Padget O, Mouritsen H, Perrins C, Guilford T (2020). Natal imprinting to the Earth’s magnetic field in a pelagic seabird. Curr Biol.

[45] Putman NF, Lohmann KJ, Putman EM, Quinn TP, Klimley AP, Noakes DLG (2013). Evidence for geomagnetic imprinting as a homing mechanism in Pacific salmon. Curr Biol.

[46] Robusto CC (1957). The Cosine-Haversine formula. Am Math Mon.

[47] European Space Agency (2021). Swarm L1b product definitions..

[48] European Space Agency (2021). VirES for Swarm.

[49] Amm O, Vanhamäki H, Kauristie K, Stolle C, Christiansen F, Haagmans R, Masson A, Taylor MGGT, Floberghagen R, Escoubet CP (2015). A method to derive maps of ionospheric conductances, currents, and convection from the Swarm multi-satellite mission. J Geophys Res Space Phys.

[50] Finlay CC, Kloss C, Olsen N, Hammer M, Toeffner-Clausen L, Grayver A, et al. The CHAOS-7 geomagnetic field model and observed changes in the South Atlantic Anomaly. Earth Planets Space. 2020;72. 10.1186/s40623-020-01252-9. 10.1186/s40623-020-01252-9 PMC7578192 33122959

[51] DTU Space (2020). The CHAOS-7 geomagnetic field model.

[52] European Space Agency (2021). Swarm L2 product definitions.

[53] Earthquakes Canada (2021). FTP server with real-time INTERMAGNET data.

[54] McLay SA, Beggan CD (2010). Interpolation of externally-caused magnetic fields over large sparse arrays using Spherical Elementary Current Systems. Ann Geophys.

[55] Beggan CD, Billingham L, Clarke E (2018). Estimating external magnetic field differences at high geomagnetic latitudes from a single station. Geophys Prospect.

[56] Schiffner I, Denzau S, Gehring D, Wiltscko R (2016). Mathematical analysis of the homing flights of pigeons based on GPS tracks. J Comp Physiol A.

[57] Kölzsch A, Müskens GJDM, Moonen S, Kruckenberg H, Glazov P, Wikelski M. Data from: Flyway connectivity and exchange primarily driven by moult migration in geese [North Sea population]: Movebank Data Repository; 2019. 10.5441/001/1.ct72m82n. 10.1186/s40462-019-0148-6 PMC6354378 30733867

[58] Kölzsch A, Müskens GJDM, Szinai P, Moonen S, Glazov P, Kruckenberg H, et al. Flyway connectivity and exchange primarily driven by moult migration in geese. Mov Ecol. 2019;7:3. 10.1186/s40462-019-0148-6. 10.1186/s40462-019-0148-6 PMC6354378 30733867

[59] Blagoveshchensky DV, Segeeva MA (2019). Impact of geomagnetic storm of September 7–8, 2017 on ionosphere and HF propagation: a multi-instrument study. Adv Space Res.

[60] Hawkes LA, Butler PJ, Frappell PB, Meir JU, Milsom WK, Scott GR (2014). Maximum running speed of captive bar-headed geese is unaffected by severe hypoxia. PLoS One.

[61] Kölzsch A, Müskens GJDM, Kruckenberg H, Glazov P, Weinzierl R, Nolet BA, Wikelski M (2016). Towards a new understanding of migration timing: slower spring than autumn migration in geese reflects different decision rules for stopover use and departure. Oikos.

[62] Jupyter Project. 2021. https://jupyter.org/. Accessed 16 Feb 2021.

[63] Smith A. ESA-VirES/VirES-Python-Client: v0.6.2 (Version v0.6.2). Zenodo; 2020. 10.5281/zenodo.3872905

[64] Clemens K. ancklo/ChaosMagPy: ChaosMagPy v0.4 (Version v0.4). Zenodo; 2020. 10.5281/zenodo.4022521

[65] Kranstauber B, Smolla M, Scharf AK (2020). move: visualizing and analyzing animal track data. R package.

[66] British Geological Survey (2021). K-indices.

[67] Welch BL (1947). The generalization of student’s problem when several different population variances are involved. Biometrika.

[68] Gastwirth JL, Gel Y, Wallace Hui QL, Lyubchich V, Miao W, Noguchi K (2020). lawstat: tools for biostatistics, public policy, and law. R package.

[69] Watson GS (1983). Statistics on spheres.

[70] Fisher NI (1995). Statistical analysis of circular data.

[71] Wallraff HG (1979). Goal-oriented and compass-oriented movements of displaced homing pigeons after confinement in differentially shielded aviaries. Behav Ecol Sociobiol.

[72] Watson GS (1962). Goodness-of-fit tests on a circle, II. Biometrika.

[73] Agostinelli C, Lund U (2017). Circular: circular statistics in R. R package.

[74] Pewsey A, Neuhäuser M, Ruxton GD, Neuhäuser M (2014). Circular statistics in R.

[75] McClintock BT, London JM, Cameron MF, Boveng PL (2015). Modelling animal movement using the Argos satellite telemetry location error ellipse. Methods Ecol Evol.

[76] Hawkes LA, Balachandran S, Batbayar N, Buttler PJ, Frappell PB, Milsom WK, Tseveenmyadag N, Newman SH, Scott GR, Sathiyaselvam P, Takekawa JY, Wikelski M, Bishop CM (2011). The trans-Himalayan flights of bar-headed geese (Anser indicus). Proc Natl Acad Sci.

[77] Chakraborty S, Morley S (2020). Probabilistic prediction of geomagnetic storms and the Kp index. J Space Weather Space Clim.

[78] European Space Agency (2021). VFM quality control reports.

