# Supplementary material for: Fusion of wildlife tracking and satellite geomagnetic data for the study of animal migration
Source: Mov Ecol. 2021 Jun 11;9:31. doi: 10.1186/s40462-021-00268-4 (PMC8196450; doi:10.1186/s40462-021-00268-4)
Supplement: Supplementary file 1 — Additional file 1. Mathematical derivations and technical details. [file 40462_2021_268_MOESM1_ESM.pdf]

Fusion of wildlife tracking and satellite geomagnetic data for the study of animal migration

Supplementary Information 1:
Mathematical derivations and technical details of the data fusion procedure

1. Great circle distance calculation

All spatial distances in our data fusion tool are great circle distances - a great circle distance is the shortest distance between two points on a sphere, where the path from one to another is located on the surface of the sphere (Figure 1).

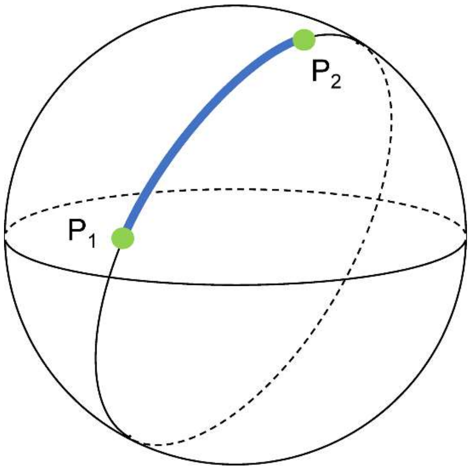

Figure 1. Diagram of the great circle distance between points P1 and P2.

Great circle distances are calculated using the haversine formula [1]. Haversine is a trigonometric function defined as:

$$\mathrm{hav}(\theta) = \sin^2\left(\frac{\theta}{2}\right) \tag{1}$$

This function is used in the calculation of the great circle distance between two points on the sphere, given with the spherical coordinates (latitude and longitude) as follows. Let P_1 have coordinates (φ_1, λ_1) and $P_2(\varphi_2, \lambda_2)$ and let Θ be the central angle between these two points. Then the haversine formula specifies the following:

$$\mathrm{hav}(\theta) = \mathrm{hav}(\phi_2 - \phi_1) + \cos(\phi_1)\cos(\phi_2)\,\mathrm{hav}(\lambda_2 - \lambda_1) \tag{2}$$

The central angle is linked to the spherical distance *d=d(P1,P2)*, or the great circle distance, as per this:

$$\theta = \frac{d}{r} \tag{3}$$

where r is the radius of the sphere. Using equations 1-3, the great circle distance d can then be calculated as:

$$d = 2r \arcsin\left(\sqrt{\sin^2\left(\frac{\phi_2 - \phi_1}{2}\right) + \cos(\phi_1)\cos(\phi_2)\sin^2\left(\frac{\lambda_2 - \lambda_1}{2}\right)}\right) \quad (4)$$

2. Implementation of our new data fusion method

In this section we provide mathematical and technical details of our implementation procedure. This description explains the code, which is provided as Supplementary Information 2 as well as available at GitHub repository MagGeo (https://github.com/MagGeo/MagGeo-Annotation-Program) or through Zenodo software repository with the following Digital Object Identifier DOI: 10.5281/zenodo.4543735.

Our method was implemented in the Python Jupyter notebooks environment [2]. Requirements to run the code are:

- Python 3.8x with the following packages installed:
- Standard spatio-temporal data science packages: Pandas, Numpy
- Specific packages for geomagnetic applications: VirES [3], chaosmagpy [4]

The implementation of our data fusion method has four steps (Figure 2):

1) From the provided GPS trajectory by the user, the program finds the list of unique dates of movement along the trajectory.

2) Using the VirES client (particularly using the Python API) where Swarm data is openly available, the program download the geomagnetic residuals for the three satellites. The residuals are the unmodeled (observed) values of the field at the altitude of the satellite. These residuals are obtained by subtracting the modelled contributions from the Core, Crust and Magnetosphere from the actual measurements.

3) A space-time interpolation process to compute magnetic residuals at the location of the GPS Point and annotate GPS trajectory with these values.

4) Calculating geomagnetic values at the location of the GPS point; this takes modelled magnetic values at the location of the GPS point (including altitude) and adds them to the magnetic residuals to create accurate geomagnetic measurements at this 3D location. Use magnetic NEC components to compute the other required magnetic values (F, I, H, D).

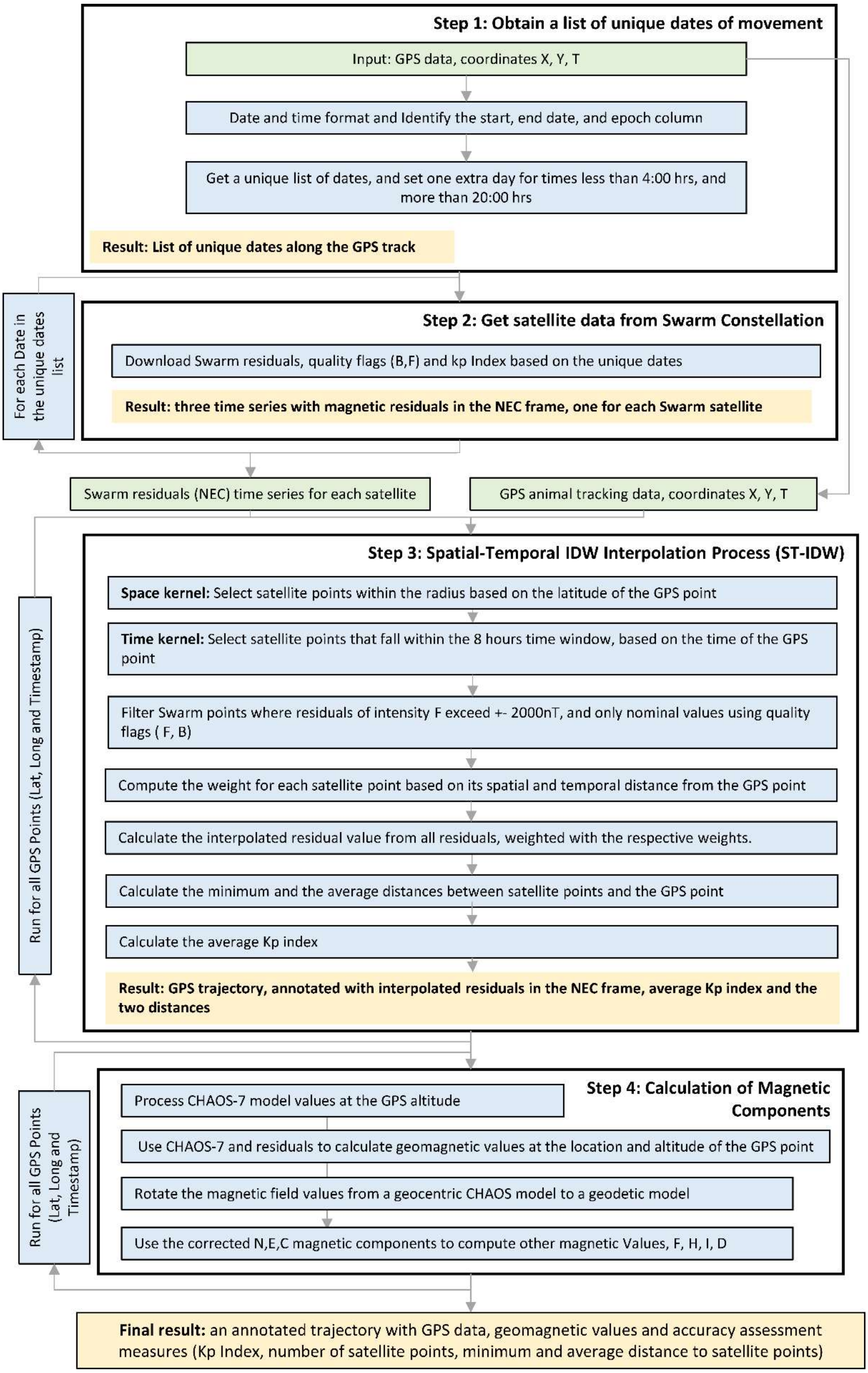

Figure 2. Flowchart of the implementation of our method.

Step 1: **<u>Obtain the list of unique dates of movement from the GPS trajectory.</u>**
In this step we import the trajectory and calculate unique dates that it covers, to optimise requests for Swarm data to the VirES client. Specifically, the trajectory has to have four fields, giving the X, Y and altitude coordinates of the tracked animal and the time T at which the location was recorded. X and Y should be longitude and latitude in decimal degrees in a geographic coordinate system (normally WGS84), to support global coverage of the method and avoid problems with having to define a local projection. From this we compute the list with unique dates that the trajectory covers.

To provide coverage for the duration of the spatio-temporal kernel (8hrs) across midnight, we add additional dates as per following. If the start point of the trajectory is before 4:00h, we add an earlier day to be able to extend the kernel backwards over midnight. If the end point of the trajectory is after 20:00h, we add the next day to be able to extend the kernel forwards over midnight.

The specific steps (as in Figure 2) are the following:

1. <u>Input</u>: GPS trajectory, which should be in a .csv file in the following format: Latitude, Longitude, Altitude, timestamp, where Latitude and Longitude are in decimal degrees in WGS84, Altitude is in kilometres above the sea level and the timestamp is in the following format: dd/mm/yyyy HH:MM:ss.
2. <u>Date and time pre-processing</u>: We compute the start and end dates and times, and the epoch column is calculated, based on the timestamp from the GPS Point. The epoch time is the number of seconds that have passed since 1 January 1970 00:00 UTC and is the usual way Python handles time. This creates new columns: Dates and Times.
3. <u>Unique list of Dates</u>: This is a function to get a list of unique dates that cover the extent of the movement data (GPS trajectories). Swarm data are organised as daily files and therefore this list is needed to request the download of Swarm data that covers all dates in the trajectory.
   In addition, to ensure that we have Swarm data from a +/- 4 hour window for all trajectory points we need to make the following check to define the correct temporal window for data download:
   - If the time of the start point of the trajectory is < 04:00:00, then we add the previous day to the Unique dates list.
   - If the time of the end point of the trajectory is > 20:00:00, then we add one further day to our list.

   Once the check is done, the resulting <u>Unique list of Dates</u> will be the input for the following stage, where Swarm data are downloaded.

Step 2: **<u>Downloading the data from Swarm (Residuals)</u>**
Swarm data are freely available through ESA's VirES client [5]. In this step we loop through the unique dates from step 1 and for each date we send a server request to obtain the following data from the VirES client:

- Locations of satellite points for all three Swarm satellites (A, B and C) on this date, these are given at 1Hz resolution and with X (longitude), Y (latitude), Z (orbital altitude) and T (timestamp) in geocentric coordinates.
- Measurements of geomagnetic field at the satellite points in the geocentric NEC system, these are observed at orbital altitude (level 1 product of Swarm data)
- We obtain the residuals values for the magnetic intensity (F) and NEC components using the CHAOS model.

- The quality flags F, B are included to further filter only the nominal measurements (that is, we only take measurements, where there were no data issues related to instrument outages, orbital manoeuvres, or satellite maintenance) [6].

For each day in the unique dates list, we request data from Swarm for all three satellites. Based on the unique dates list, the script will download for each day in a 24-hour period the Level 1b and Level 2 Swarm data from VirES-Python-Client [5] for all three satellites A, B and C. To get access to the data using the python client a web token is required to granted permissions over VirES for Swarm. For more details on this see the code.

For each satellite, the function Get_Swarm_residuals function will extract the following information:

1. A time series of satellite locations for each satellite for a period of 24 hours, including the Latitude, Longitude, and timestamp values at 1Hz resolution (this is 24 hours of data, so 86,400 records (one per second)).
2. A time series of the magnetic measurements 'B_NEC', which is a three-dimensional vector with magnetic measurements in the NEC coordinate system at the location and time of the satellite, and 'F_Res' which is the reported magnetic intensity residuals. This F_res values will be used to filter Swarm points reporting not nominal values for the intensity residuals.
3. The magnetic measurements are the Swarm residuals, which are the unmodeled magnetic values at the satellite altitude, after modelled contribution from the core, the crust and the magnetosphere have been removed from the raw measurements. The residuals that we obtain in this way represent the real-time temporal variation induced by the solar wind and exclude effects of both the Earth's internal fields and the fields in the magnetosphere (excluding ionospheric effects).
4. The quality FlagsB and FlagsF requested in the data, are now included to filter the swarm points to use only the nominal measures related to the magnetic field intensity and the magnetic field vector measurements [6].
5. In the final step we organise these data in a pandas data frame – this now includes the magnetic residuals (NEC), F residuals, quality flags, latitude, longitude, and time for each Swarm satellite point.

This process is executed for each unique date in the list and creates one data set for each satellite. The result of this step are three time series (one per satellite) of satellite and geomagnetic measurements for the requested dates in a 24-hours period:

- TotalSwarm_A
- TotalSwarm_B
- TotalSwarm_C

Step 3: Spatio-temporal IDW interpolation (ST-IDW) and annotation.

In this step we run a loop across all trajectory points. For each point we select satellite points that fall within the respective space-time kernel. We calculate spatio-temporal weights for each satellite point and interpolate the geomagnetic NEC residuals values using these weights. We also calculate measures for accuracy assessment: the number of total satellite points for each trajectory point and the minimum and average distance from the trajectory point to the set of respective satellite points. An extra attribute to help users identify the global average magnetic disturbance for the position and time of each GPS point is also included as the average Kp index.

The result is an annotated GPS trajectory, where for each GPS location and time we have a set of geomagnetic residuals values (N, E, C) and accuracy assessment measures (distances and Kp index).

The specific steps (as in Figure 2) are the following:

Step 3.1: Spatio-temporal kernel
This process starts by calculating a spatio-temporal kernel to select selection of satellite points used for interpolation for one trajectory point. This step is run for each trajectory point and consists of the following two steps:

1. Calculating the time-kernel:
Let trajectory point be given as *P (X, Y, Z, T)*, where X is the longitude, Y the latitude, Z the altitude and T the time at which it was collected.

Time kernel: select all points from the Swarm data frame that are within +-4 hours of the given trajectory point. DfTime_func is the function that implements this time filter.

2. Calculating the space-kernel:
The kernel uses the great circle distance, which does not include altitude.
There are two functions to compute this kernel (distance_to_GPS and Kradius)

We first calculate the great circle distance ds_i between all satellite points in the time kernel selection and the trajectory point using the haversine formula (d in eq. 4, implemented as distance_to_GPS function).

We the use Kradius function to calculate the radius *r* of the circle within which we will be looking for the satellite points. This radius is based on the latitude of the trajectory point and is calculated so that it reaches 1800 kilometres at the Equator and 900km at the poles (Figure 5A in the main paper). For source of these numbers see the main paper. The radius is given by the following equations:

For northern latitudes (Y >= 0):

$$r = -10 * Y + 1800 \tag{5}$$

For southern latitudes (Y < 0):

$$r = 10 * Y + 1800 \tag{6}$$

Once the radius and the distances are calculated, we select all satellite points from the time kernel selection where: $ds_i <= r$.

Step 3.2: Filter selected Swarm measurements by nominal values only.

This process filters out the "bad" Swarm points collected in Step 2. There are two filters: initially, all Swarm points included in the spatio-temporal kernel (Step 3.1) will be filtered by intensity residuals values(F_res) that exceed +-2000 nT. It is extermely rare to have a variation of more than 2000 nT of the ionoshperic field – this generally happens less than once per decade [7]. Such large values therefore indicate a problem with the Swarm data and can be used for elimination of problematic values. Based on table 15 in [6], there is a second filter to select only nominal measurements. We filter the Swarm point to those only with Flags_F and Flags_B = 0, representing only nominal measurements, that is those measurements where there were no technical or maintenance issues with either the satellites or the instruments [6].

Step 3.3: Inverse Distance Weighted Interpolation (ST-IDW).

This process is shown in Figure 5B in the main paper. For each of the *n* selected satellite points $S_i(Xs_i, Ys_i, Ts_i)$, $i=1,\ldots,n$ we calculate the absolute temporal difference dt_i, $i=1,\ldots,n$ from the trajectory point *P(X,Y,T)*. Here X and Xs*i* are the latitudes of the trajectory point and all the satellite points, Y are the longitudes and T are respective times. This is done by:

$$dt_i = | T_{Si} - T |, \qquad i = 1, \ldots, n \tag{7}$$

We already have the great circle distance ds_i, $i=1,\ldots,n$, between each selected satellite point and the trajectory point.

For the IDW interpolation, we wish to give equal weights to both the spatial and temporal distance and prioritise satellite points that are close to the trajectory point in either space or time (Figure 5B in the main paper). Therefore, if the satellite is directly above the trajectory point, we want the weight to reflect the temporal distance only. Similarly, if the satellite points are measured at the same time as the trajectory point but are in different locations, we want the weight to reflect the spatial distance only. We also need to account for the different scales in which temporal and spatial locations are measured and therefore need to normalise the two distances.

To achieve this, we define normalised versions of each temporal and spatial distance. The normalised spatial distance for a satellite point *i*, A_i, is given as:

$$A_i = 1 - \frac{r - ds_i}{r} = \frac{ds_i}{r}, \qquad i = 1, \ldots, n \tag{8}$$

Here, ds_i is the great circle distance between the trajectory point and the satellite point *i* and *r* is the radius that defines the space kernel (eq. 5 or 6).

Similarly, the normalised temporal distance for a satellite point *i*, B_i, is given as:

$$B_i = 1 - \frac{\Delta T - dt_i}{\Delta T} = \frac{dt_i}{\Delta T}, \qquad i = 1, \ldots, n \tag{9}$$

Here, ΔT is the half size of the temporal window that defines the time-kernel (in our case $\Delta T = 4hr$) and dt_i is the absolute temporal distance between the trajectory point and the satellite point *i*.

To join the two, we define a combined normalised distance d_i, as per following (function DistJ):

$$d_i = \sqrt{\frac{{A_i}^2 + {B_i}^2}{2}}, \qquad i = 1, \dots, n \tag{10}$$

Since A_i and B_i are both scaled from 0 to 1, this normalisation also scales d_i between 0 and 1, giving the highest values at furthest distances in both space and time.

Now we can use the Inverse Distance Weighted Interpolation (IDW) formula to create weights w_i that prioritise the closest satellite points in space and time and do so for all satellite points $i=1,\dots,n$. This is computed in the ST_IDW_Process function that will at the same time invoke the previous functions.

Let d_i $i=1,\dots n$, be the combined normalised distance from the trajectory point to the selected satellite points from Swarm. We calculate the interpolation weights in two steps, first we calculate inverse distances as $w1_i$:

$$w1_i = \frac{1}{d_i^2}, \qquad i = 1, \dots, n \tag{11}$$

Weights are then normalised, so that they sum up to 1:

$$w_i = \frac{w1_i}{\sum_{i=1}^{n} w_{1i}}, \qquad i = 1, \dots, n \tag{12}$$

Interpolated residuals measures (NEC) at the location of the trajectory point are then calculated as follows:

$$N_{res} = \sum_{i=1}^{n} w_i * N_{res\_i} \tag{13}$$

$$E_{res} = \sum_{i=1}^{n} w_i * E_{res\_i} \tag{14}$$

$$C_{res} = \sum_{i=1}^{n} w_i * C_{res\_i} \tag{15}$$

Here, N_{res} is the interpolated value of the residual in the direction of N axis at the location of the trajectory point, w_i is the weight to i-th satellite point (eq. 12) and N_{res_i} is the residual of the magnetic field in the direction of N axis at the i-th satellite point at the same moment in time as the trajectory point. Equations 14 and 15 give the same calculation for measurements in the E and C axes.

Step 3.4. Calculate accuracy measures.

For accuracy assessment we need the total number of satellite points that were used for interpolation at each trajectory point. This has already been counted above as n and is now appended to the GPS trajectory.

In addition to this, there are two further attributes for the accuracy measurement that the method will compute, the minimum and average spatial distance between the GPS point and its respective selected satellite points ($avgD$: AvDistance, *minD*: MinDistance):

$$avgD = \frac{1}{n} * \sum_{i=1}^{n} ds_i , i = 1, \ldots n \quad \text{and} \quad minD = \min(d_i) , i = 1, \ldots , n \qquad (16)$$

Step 3.5. Calculate the average local Kp index.

Using the Kp index values requested from VirES, we compute the average local Kp index values for each GPS point. This value helps to identify when there is high magnetic disturbance at the location and time of each GPS point. If the Kp index value is from 0 to 2 it is a quiet period of little or no magnetic disturbance. From 3 up to 6 there is a medium or moderate magnetic disturbance, and from 7 to 9 the Kp index describes strong magnetic activity caused by an intensive storm. For more information about the Kp index see the main paper. We calculate the average local Kp index using Kp values from all satellite points:

$$avgKpIndex = \frac{1}{n} * \sum_{i=1}^{n} kp_i , i = 1, \ldots n \qquad (17)$$

Finally, we annotate the trajectory point with the following new attributes: the interpolated geomagnetic residuals values (N_{res}, E_{res}, C_{res}), the accuracy assessment measures (*n, avgD, minD*) and the average local Kp index value. This is implemented as the return of the ST_IDW_Process function.

Step 4: Calculate the geomagnetic componentsfor the annotated GPS track.

Once the GPS track was annotated with the interpolated magnetic residuals (N_{res}, E_{res}, C_{res}), we calculate the geomagnetic measures at the altitude of the GPS point. This is done by the two following steps:

4.1 To calculate magnetic values (N,E,C) at the altitude of the GPS trajectory, we use the CHAOS-7 model to compute the Crust, Core and Magnetosphere contributions at the GPS Altitude level. If there is no altitude in the GPS data, our method will use the WGS-84 ellipsoid (0 km) as the altitude value. CHAOS-7 model provides values of different field components directly, but for more on how geophysicists disaggregate the total magnetic field into its components using spherical harmonical analysis, see [8].

The CHAOS model uses a Geocentric reference frame with Phi as the colatitude, Theta as longitude, and Radial value as the combination of the Earth's radius and GPS track altitude in kilometres. We

use the CHAOS-7 model and the previous parameters to compute the Core, Crust and Magnetosphere contributions in Phi, Theta and Radial components. This difference of reference frame involves a transformation between the geocentric NEC frame from the satellite data and the geodetic NEC frame (WGS84) from the GPS trajectory. This step also requires a rotation between both reference frame, that transformation is apply using the gg_to_geo function. For more information about the equation used in this reference frame rotation, see [9-10].

4.2 Once the CHAOS magnetic components are computed (Phi, Theta, and Radial), we add the Swarm residuals to calculate geomagnetic values at the 3D location of the GPS point. This effectively translates the measured short-term solar wind induced variation and ionospheric disturbance (neither of which can be modelled and can only be measured) to the longer-term modelled values. The result are geomagnetic components at the X, Y, Z and T of GPS point. This is implemented as the return of the CHAOS_ground_values function.

Steps 4.1. to 4.2. are repeated in a loop across all trajectory points, to obtain the final results as GPS_ResInt data frame which is the annotated trajectory with the following attributes: Latitude, Longitude, Altitude timestamp, *N*, *E*,*C*, *Kp*, *n, avgD, minD*.

Step 4.1: Calculate other geomagnetic components at the trajectory point.

Once we have the three accurate magnetic values in the NEC directions, we rotate them into the WGS-84 geodetic frame and calculate the values of intensity F, length of the horizontal component H, declination D and inclination I using the following formulas (Figure 1B in the main paper). The subscript int indicates that these are calculated from the interpolated values.

$$\mathrm{F} = \sqrt{\mathrm{N}^2 + \mathrm{E}^2 + \mathrm{C}^2} \tag{18}$$

$$H = \sqrt{\mathrm{N}^2 + \mathrm{E}^2} \tag{19}$$

$$D = \arctan\left(\frac{E}{N}\right) \tag{20}$$

$$I = \arctan\left(\frac{C}{H}\right) \tag{21}$$

Once the additional magnetic components are computed, the final data frame has in WGS-84 coordinates: Latitude, Longitude, Altitude, timestamp, *N*, *E*,*C*, *Kp*, *n, avgD, minD, H, F, I and D* attributes. The data frame can be exported as a CSV file and used in further analysis of migratory behaviour.

References

1. Robusto CC. 1957. The Cosine-Haversine Formula. *The American Mathematical Monthly* **64**(1), 38-40. (doi: 10.2307/2309088)
2. Jupyter Project. 2014 https://jupyter.org/ (Last accessed 11 September 2020)
3. Ashley Smith, & pacesm. (2020, September 16). ESA-VirES/VirES-Python-Client: v0.7.0 (Version v0.7.0). Zenodo. http://doi.org/10.5281/zenodo.4032884 (last accessed 20 February 2021)
4. Clemens Kloss. (2020). ancklo/ChaosMagPy: ChaosMagPy v0.4 (Version v0.4). Zenodo. http://doi.org/10.5281/zenodo.4022521(last accessed 20 February 2021)
5. European Space Agency (ESA). 2020 *VirES for Swarm*. https://vires.services/ (last accessed 7 June 2020)
6. European Space Agency (ESA). Characterization Flags of the magnetic field intensity measurement and vector measurement. https://earth.esa.int/web/guest/missions/esa-eo-missions/swarm/data-handbook/level-1b-product-definitions#label-Flags_F-and-Flags_B-Values-of-MDR_MAG_LR. (last accessed 20 April 2021)
7. Beggan, C.D., Billingham, L. and Clarke, E. (2018), Estimating external magnetic field differences at high geomagnetic latitudes from a single station. Geophysical Prospecting, 66: 1227-1240. https://doi.org/10.1111/1365-2478.12641
8. Campbell WH. 2003 *Introduction to geomagnetic fields*. Cambridge University Press, 2nd edition.
9. Jacobs JA, Jacobs JA, Filloux JH. 1987 Geomagnetism. Academic Press, the University of California.
10. Malin SRC, Barraclough DR. 1981 An algorithm for synthesizing the geomagnetic field*. Computers and Geosciences* **7**(4):401-405. (doi: 10.1016/0098-3004(81)90082-0)
