# Supplementary material for: Fusion of wildlife tracking and satellite geomagnetic data for the study of animal migration
Source: Mov Ecol. 2021 Jun 11;9:31. doi: 10.1186/s40462-021-00268-4 (PMC8196450; doi:10.1186/s40462-021-00268-4)

MagGeo - Sequential Mode

April 26, 2021

1 MagGeo - Sequential Mode

Authors | Fernando Benitez-Paez, Urška Demšar, Jed Long, Ciaran Beggan

Contact | Fernando.Benitez@st-andrews.ac.uk, ud2@st-andrews.ac.uk, jed.long@uwo.ca, ciar@bgs.ac.uk

Keywords | Bird migration, data fusion, Earth's magnetic field, Swarm, GPS tracking

1.1 Overview

This Jupyter Notebook will guide you through the required steps to annotate your GPS tracking data with the earth's magnetic field data from Swarm (European Space Agency). This version is called Sequential Mode, alternatively you can use Parallel Mode to take advantage of parallelized computing if required. More information about the Swarm satellites can be found in the Main Document on the MagGeo github repository. This script will use a sequential loop to run the annotation process for each GPS Point (row) from your data.

To execute the code, you can go through each cell (pressing Crtl+Enter), you will also find inner comments **##** to describe each particular step. If you are not familiar with using Jupyter Notebooks, you might want to take some time to learn how first, for example take a look at the notebook-basics.ipynb Notebook inside MagGeo.

1.2 Data requirements

Your trajectory must be in a csv format:

There are three columns that must be included in your GPS trajectory. Make sure your GPS trajectory includes **Latitude** , **Longitude** and **timestamp**. We suggest that the Timestamp column follow the day/month/year Hour:Minute (**dd/mm/yyyy HH:MM:SS**) format, Latitude and Longitude should be in decimal degrees (WGS84). Optionally an altitude column can be used providing altitude (the altitude must be in **km**). Other Columns will be ignored. Here it is an example of how your GPS track should look:

For this example we are reading the BirdGPSTrajectory.csv file. If you want to run the method using your own csv file, make sure you store your the file in the ./data folder. For more information about the dataset we used in this example go to the Main Notebook.

1.3 Import the required python libraries

[1]:
```
import datetime as dt
import sys,os
from pathlib import Path
import pandas as pd
import numpy as np
import datetime, time
from datetime import timedelta
import math
import pathlib
from datetime import datetime
import time
import calendar
import datetime
from viresclient import ClientConfig
import matplotlib.pyplot as plt
from viresclient import set_token
from MagGeoFunctions import getGPSData
from MagGeoFunctions import Get_Swarm_residuals
from MagGeoFunctions import ST_IDW_Process
from MagGeoFunctions import CHAOS_ground_values
```

1.4 Add your VirES web client Token

The **VirES client API**, requires a token. Before start you need to get your own VirES token. You can visit https://vires.services/ to get yours, and then add it into the next cell.

[2]:
```
set_token("https://vires.services/ows", set_default=True)
```

```
Enter token:········
Token saved for https://vires.services/ows
```

1.5 Read the GPS track

The following steps will load the GPS track from a csv file, and set some requirements before downloading geomagnetic data from Swarm. If your csv track file doesnt not have any altitude attribute, MagGeo will use sea level as your altitude (i.e., 0 Km). **Altitude column units must be Km**

[3]:
```
#Make sure the csv file of your trackectory is stored in the Data folder.
#Enter the name of your GPS track csv file including the extension .csv  and␣
↪press Enter (e.g. BirdGPSTrajectory.csv)
os.chdir(r"./data")
gpsfilename=input("What is the name of your .csv file?: ") # i.e␣
↪BirdGPSTrajectory.csv
Lat=input("Enter the name of your Latitude column?: ") #i.e location-lat
Long=input("Enter the name of your Longitud column?: ") # i.e location-long
```

```
DateTime=input("Enter the date and time column name?: ") # i.e timestamp
altitude = input("Enter the Altitude column name?, if you don't have the␣
 ↪altitude column, just press Enter: ")
# i.e height (Only in KM)
#If your csv track file doesnt not have any altitude attribute, MagGeo will use␣
 ↪sea level as your altitude (i.e. 0 Km).
```

```
What is the name of your .csv file?: BirdGPSTrajectory.csv
Enter the name of your Latitude column?: location-lat
Enter the name of your Longitud column?: location-long
Enter the date and time column name?: timestamp
Enter the Altitude column name?, if you don't have the altitude column, just
press Enter: height
```

[4]:
```
# Here MagGeo is reading your CSV file, taking the Lat, Long, Date&Time and␣
 ↪Altitutes attributes and compute, some aditional attrubutes we need to the␣
 ↪annotation process.
# Setting the date and time attributes for the required format and computing␣
 ↪the epoch column. Values like Maximum and Minimun Date and time are also␣
 ↪calculated.
GPSData = getGPSData(gpsfilename,Lat,Long,DateTime,altitude)
os.chdir(r"../")
GPSData
```

[4]:
```
             gpsDateTime    gpsLong     gpsLat  gpsAltitude       epoch  \
0    2014-09-08 05:54:00  68.307333  70.854717        0.000  1410155640
1    2014-09-08 06:10:00  67.975050  70.830300        0.406  1410156600
2    2014-09-08 06:26:00  67.752417  70.761717        0.498  1410157560
3    2014-09-08 06:42:00  67.561983  70.686517        0.787  1410158520
4    2014-09-08 07:14:00  67.548317  70.685450        0.337  1410160440
..                   …          …          …          …           …
968  2014-10-01 10:37:00  22.509733  55.214750        0.191  1412159820
969  2014-10-03 11:28:00   7.292517  53.392167        0.184  1412335680
970  2014-10-04 07:11:00   5.922067  53.107067        0.181  1412406660
971  2014-10-04 14:12:00   5.811383  53.014967        0.190  1412431920
972  2014-10-04 21:05:00   5.729433  53.009167        0.193  1412456700

          dates     times
0    2014-09-08  05:54:00
1    2014-09-08  06:10:00
2    2014-09-08  06:26:00
3    2014-09-08  06:42:00
4    2014-09-08  07:14:00
..          …         …
968  2014-10-01  10:37:00
969  2014-10-03  11:28:00
970  2014-10-04  07:11:00
```

```
971  2014-10-04  14:12:00
972  2014-10-04  21:05:00

[973 rows x 7 columns]
```

1.6 Validate the correct amount of Swarm measures

The following loop is identifiying the time and validating if the time is less than 4:00 hours and more than 20:00 hours to bring one extra day of data. The result of this validation is written in an empty python list which will be later validated to get the unique dates. This avoids duplicate downloading of data for the same day and reduces overall computational time.

[5]:
```
datestimeslist = []
for index, row in GPSData.iterrows():
    datetimerow  = row['gpsDateTime']
    daterow = row['dates']
    hourrow = row['times']
    hourrow = hourrow.strftime('%H:%M:%S')
    if hourrow < '04:00:00':
        date_bfr = daterow - (timedelta(days=1))
        datestimeslist.append(daterow)
        datestimeslist.append(date_bfr)
    if hourrow > '20:00:00':
        Date_aft = daterow + (timedelta(days=1))
        datestimeslist.append(daterow)
        datestimeslist.append(Date_aft)
    else:
        datestimeslist.append(daterow)
```

Getting a list of unique dates to download the Swarm Data

[6]:
```
def uniquelistdates(list):
    x = np.array(list)
    uniquelist = np.unique(x)
    return uniquelist

uniquelist_dates = uniquelistdates(datestimeslist)
uniquelist_dates
```

[6]:
```
array([datetime.date(2014, 8, 18), datetime.date(2014, 8, 19),
       datetime.date(2014, 8, 25), datetime.date(2014, 8, 26),
       datetime.date(2014, 8, 27), datetime.date(2014, 8, 28),
       datetime.date(2014, 8, 29), datetime.date(2014, 8, 30),
       datetime.date(2014, 8, 31), datetime.date(2014, 9, 3),
       datetime.date(2014, 9, 5), datetime.date(2014, 9, 6),
       datetime.date(2014, 9, 8), datetime.date(2014, 9, 13),
       datetime.date(2014, 9, 14), datetime.date(2014, 9, 15),
```

```
 datetime.date(2014, 9, 17), datetime.date(2014, 9, 18),
 datetime.date(2014, 9, 19), datetime.date(2014, 9, 20),
 datetime.date(2014, 9, 21), datetime.date(2014, 9, 22),
 datetime.date(2014, 9, 23), datetime.date(2014, 9, 24),
 datetime.date(2014, 9, 25), datetime.date(2014, 9, 26),
 datetime.date(2014, 9, 27), datetime.date(2014, 9, 29),
 datetime.date(2014, 9, 30), datetime.date(2014, 10, 1),
 datetime.date(2014, 10, 2), datetime.date(2014, 10, 3),
 datetime.date(2014, 10, 4), datetime.date(2014, 10, 5),
 datetime.date(2014, 10, 6), datetime.date(2014, 10, 10),
 datetime.date(2014, 10, 13), datetime.date(2014, 10, 14),
 datetime.date(2014, 10, 15), datetime.date(2014, 10, 22),
 datetime.date(2014, 10, 23), datetime.date(2014, 10, 24)],
dtype=object)
```

1.7 Download Swarm residuals data

Once the date and time columns have been defined and the unique dates are identified the script can start the download process. Usually the data from Swarm is requested using only one satellite, however **MagGeo** will use the magnetic measures from the three satellite of the Swarm Mission (Alpha, Bravo, Charlie). Be aware satellite Charlie, got its AMS broken earlier in the mission, although the initial dates still have valid data MagGeo can use.

Be aware: Due to the amount of dates in the demo GPS track (42 days), the time to process the sample data will take approximately 10 minutes. Unfortunatly the download process migth be a slow process, particually for the magnetic models data MagGeo requieres.

Set a connection to the VirES client and using the function Get_Swarm_residuals we will get the swarm residuals for the dates included in the previous list.

[ ]:
```
%%time

hours_t_day = 24 #MagGeo needs the entire Swarm data for each day of the␣
↪identified day.
hours_added = datetime.timedelta(hours = hours_t_day)

listdfa = []
listdfb = []
listdfc = []

for d in uniquelist_dates:
    print("Getting Swarm data for date:",d )
    startdate = datetime.datetime.combine(d, datetime.datetime.min.time())
    enddate = startdate + hours_added
    SwarmResidualsA,SwarmResidualsB,SwarmResidualsC =␣
↪Get_Swarm_residuals(startdate, enddate)
    listdfa.append(SwarmResidualsA)
    listdfb.append(SwarmResidualsB)
```

```
    listdfc.append(SwarmResidualsC)
```

Concat the previous results and temporally save the requested data locally: Integrate the previous list for all dates, into pandas dataframes. We will temporally saved the previous results, in case you need to re-run MagGeo, with the following csv files you will not need to run the download process.

[8]:
```
%%time
os.chdir(r"./temp_data")
TotalSwarmRes_A = pd.concat(listdfa, join='outer', axis=0)
TotalSwarmRes_A.to_csv ('TotalSwarmRes_A.csv', header=True)
TotalSwarmRes_B = pd.concat(listdfb, join='outer', axis=0)
TotalSwarmRes_B.to_csv ('TotalSwarmRes_B.csv', header=True)
TotalSwarmRes_C = pd.concat(listdfc, join='outer', axis=0)
TotalSwarmRes_C.to_csv ('TotalSwarmRes_C.csv', header=True)
os.chdir(r"../")
TotalSwarmRes_A #If you need to take a look of the Swarm Data, you can print␣
↪TotalSwarmRes_B, or TotalSwarmRes_C
```

```
CPU times: user 4.04 s, sys: 183 ms, total: 4.22 s
Wall time: 4.26 s
```

[8]:

	F_res	Kp	Latitude	Longitude	Flags_F	Radius	\
epoch							
1408320000	6.172334	1.0	66.843847	-134.672531	1	6832723.53	
1408320030	5.200886	0.7	68.750719	-134.175850	1	6832542.55	
1408320060	2.146256	0.7	70.654918	-133.562740	1	6832377.42	
1408320090	-2.443055	0.7	72.555551	-132.795266	1	6832228.58	
1408320120	-7.002322	0.7	74.451293	-131.817227	1	6832096.43	
…	…	…	…	…	…		
1414195050	3.577492	1.7	-31.747197	126.692648	1	6846883.18	
1414195080	5.393771	1.7	-29.835345	126.687015	1	6846698.22	
1414195110	6.468472	1.7	-27.923204	126.676892	1	6846503.98	
1414195140	6.738029	1.7	-26.010783	126.662769	1	6846300.35	
1414195170	6.425266	1.7	-24.098091	126.645088	1	6846087.25	

	Spacecraft	Flags_B	N_res	E_res	C_res	\
epoch						
1408320000	A	0	-2.784954	9.087620	6.157086	
1408320030	A	0	-16.267735	24.676547	6.178650	
1408320060	A	0	-29.700036	34.292158	4.071577	
1408320090	A	0	-37.796721	44.267360	-0.604402	
1408320120	A	0	-72.888506	62.526620	-3.285812	
…	…	…	…	…	…	
1414195050	A	0	-9.562833	6.730607	-8.331892	
1414195080	A	0	-7.087156	5.130584	-9.583105	
1414195110	A	0	-4.444862	4.759146	-9.769941	

```
1414195140           A        0  -2.557345   1.961955 -9.392708
1414195170           A        0  -1.458364   0.887033 -8.652785

                     timestamp
epoch
1408320000 2014-08-18 00:00:00
1408320030 2014-08-18 00:00:30
1408320060 2014-08-18 00:01:00
1408320090 2014-08-18 00:01:30
1408320120 2014-08-18 00:02:00
…                           …
1414195050 2014-10-24 23:57:30
1414195080 2014-10-24 23:58:00
1414195110 2014-10-24 23:58:30
1414195140 2014-10-24 23:59:00
1414195170 2014-10-24 23:59:30

[120960 rows x 12 columns]
```

1.8 Spatio-Temporal filter and interpolation process (ST-IDW)

Once we have requested the swarm data, now we need to `filter` in space and time the available points to compute the magnetic values (NEC frame) for each GPS point based on its particular date and time. The function ST_IDW_Process takes the GPS track and the downloaded data from swarm to filter in space and time based on the criteria defined in our method. With the swarm data filtered we interpolate (IDW) the NEC components for each GPS data point.

[ ]:
```
%%time
#Sequential mode, applying a traditional loop using iterrows.
if __name__ == '__main__':
    dn = [] ## List used to add all the GPS points with the annotated MAG Data.␣
 ↪See the last bullet point of this process
    for index, row in GPSData.iterrows():
        GPSLat = row['gpsLat']
        GPSLong = row['gpsLong']
        GPSDateTime = row['gpsDateTime']
        GPSTime = row['epoch']
        GPSAltitude = row['gpsAltitude']
        print("Process for:", index,"DateTime:",GPSDateTime)
        try:
            result=ST_IDW_Process(GPSLat,GPSLong,GPSAltitude,␣
 ↪GPSDateTime,GPSTime, TotalSwarmRes_A, TotalSwarmRes_B, TotalSwarmRes_C)
            dn.append(result)
        except:
            print("Ups!.That was a bad Swarm Point, let's keep working with the␣
 ↪next point")
```

```
            result_badPoint= {'Latitude': GPSLat, 'Longitude': GPSLong,␣
↪'Altitude':GPSAltitude, 'DateTime': GPSDateTime, 'N_res': np.nan, 'E_res':␣
↪np.nan, 'C_res':np.nan, 'TotalPoints':0, 'Minimum_Distance':np.nan,␣
↪'Average_Distance':np.nan}
            dn.append(result_badPoint)
            continue
```

Temporally save the ST-IDW result locally. Still MagGeo needs to run the calculation of geomagnetic components, brining the magnetic values at the altitude provided for your GPS track.

[10]:
```
os.chdir(r"./temp_data")
GPS_ResInt = pd.DataFrame(dn)
GPS_ResInt.to_csv ('GPS_ResInt.csv', header=True)
os.chdir(r"../")
GPS_ResInt
```

[10]:
```
      Latitude  Longitude  Altitude             DateTime      N_res      E_res  \
0    70.854717  68.307333     0.000  2014-09-08 05:54:00 -14.762849   9.940227
1    70.830300  67.975050     0.406  2014-09-08 06:10:00 -14.258814   9.265709
2    70.761717  67.752417     0.498  2014-09-08 06:26:00 -11.272273   7.400717
3    70.686517  67.561983     0.787  2014-09-08 06:42:00 -10.660880   6.692922
4    70.685450  67.548317     0.337  2014-09-08 07:14:00  -9.508182   5.531622
..         …          …        …                    …          …          …
968  55.214750  22.509733     0.191  2014-10-01 10:37:00   0.495444  -1.222250
969  53.392167   7.292517     0.184  2014-10-03 11:28:00   5.011037  -1.366450
970  53.107067   5.922067     0.181  2014-10-04 07:11:00  -2.740260  -6.233470
971  53.014967   5.811383     0.190  2014-10-04 14:12:00   3.898109 -11.085052
972  53.009167   5.729433     0.193  2014-10-04 21:05:00   1.212779  -2.286258

         C_res  TotalPoints  Minimum_Distance  Average_Distance        Kp
0     1.286485           46        327.950987        665.008368  1.308696
1     1.367861           46        340.038476        667.146029  1.308696
2     1.148672           55        348.223318        678.815409  1.190909
3     1.207128           55        355.472899        680.040733  1.190909
4     1.269740           55        355.980432        680.062802  1.190909
..         …            …               …                 …         …
968  -5.801082           45        240.371734        825.618960  2.406667
969  -7.475049           40        100.997905        755.121540  0.300000
970  -6.999515           24        352.394408        784.016294  1.000000
971 -10.503966           21        179.979519        889.678639  1.000000
972   0.562593           50        100.916767        883.585143  1.700000

[973 rows x 11 columns]
```

1.9 Compute the magnetic components at the trajectory altitute using CHAOS model

The function CHAOS_ground_values is used to run the calculation of magnetic components. This adjustment requeries the magnetic components at the trajectory altitude (or at the ground level) using CHAOS (theta, phi, radial). This process also further conducts the rotation and transformation between a geocentric earth-based reference system (CHAOS) and geodetic earth-based reference system (GPS track). Once the corrected values are calculated the non-necesary columns are removed. For more information about this process go to the Main Notebook.

[11]:
```
%%time
X_obs, Y_obs, Z_obs =CHAOS_ground_values(GPS_ResInt)
GPS_ResInt['N'] =pd.Series(X_obs)
GPS_ResInt['E'] =pd.Series(Y_obs)
GPS_ResInt['C'] =pd.Series(Z_obs)
GPS_ResInt.drop(columns=['N_res', 'E_res','C_res'], inplace=True)
GPS_ResInt
```

```
CPU times: user 1.4 s, sys: 153 ms, total: 1.55 s
Wall time: 1.59 s
```

[11]:
```
      Latitude  Longitude  Altitude            DateTime  TotalPoints  \
0    70.854717  68.307333     0.000 2014-09-08 05:54:00           46
1    70.830300  67.975050     0.406 2014-09-08 06:10:00           46
2    70.761717  67.752417     0.498 2014-09-08 06:26:00           55
3    70.686517  67.561983     0.787 2014-09-08 06:42:00           55
4    70.685450  67.548317     0.337 2014-09-08 07:14:00           55
..         …          …        …                   …          …
968  55.214750  22.509733     0.191 2014-10-01 10:37:00           45
969  53.392167   7.292517     0.184 2014-10-03 11:28:00           40
970  53.107067   5.922067     0.181 2014-10-04 07:11:00           24
971  53.014967   5.811383     0.190 2014-10-04 14:12:00           21
972  53.009167   5.729433     0.193 2014-10-04 21:05:00           50

     Minimum_Distance  Average_Distance        Kp             N            E  \
0          327.950987        665.008368  1.308696   6949.105566  3851.158789
1          340.038476        667.146029  1.308696   6985.653894  3866.337781
2          348.223318        678.815409  1.190909   7035.551690  3877.443099
3          355.472899        680.040733  1.190909   7082.978312  3886.943680
4          355.980432        680.062802  1.190909   7086.091085  3888.485656
..                …                 …         …             …            …
968        240.371734        825.618960  2.406667  16968.785159  1828.024623
969        100.997905        755.121540  0.300000  18345.670851   502.789527
970        352.394408        784.016294  1.000000  18525.835109   337.275930
971        179.979519        889.678639  1.000000  18582.545311   321.253970
972        100.916767        883.585143  1.700000  18585.390848   318.935806

                C
```

```
0      57703.400422
1      57645.097979
2      57609.988412
3      57575.069804
4      57584.964457
..              …
968    47875.310888
969    45904.683940
970    45661.727719
971    45603.873424
972    45607.600808

[973 rows x 11 columns]
```

1.10 The final result

With the NEC components for each GPS Track point, it is possible to compute the aditional magnetic components. For more information about the magnetic components and their relevance go to the main paper or notebook.

[12]:
```
%%time
# Having Intepolated and weighted the magnetic values, we can compute the other␣
↪magnectic components.
GPS_ResInt['H'] = np.sqrt((GPS_ResInt['N']**2)+(GPS_ResInt['E']**2))
#check the arcgtan in python., From arctan2 is saver.
DgpsRad = np.arctan2(GPS_ResInt['E'],GPS_ResInt['N'])
GPS_ResInt['D'] = np.degrees(DgpsRad)
IgpsRad = np.arctan2(GPS_ResInt['C'],GPS_ResInt['H'])
GPS_ResInt['I'] = np.degrees(IgpsRad)
GPS_ResInt['F'] = np.
↪sqrt((GPS_ResInt['N']**2)+(GPS_ResInt['E']**2)+(GPS_ResInt['C']**2))
GPS_ResInt
```

```
CPU times: user 3.93 ms, sys: 1.07 ms, total: 5 ms
Wall time: 4.01 ms
```

[12]:
```
      Latitude  Longitude  Altitude            DateTime  TotalPoints  \
0    70.854717  68.307333     0.000 2014-09-08 05:54:00           46
1    70.830300  67.975050     0.406 2014-09-08 06:10:00           46
2    70.761717  67.752417     0.498 2014-09-08 06:26:00           55
3    70.686517  67.561983     0.787 2014-09-08 06:42:00           55
4    70.685450  67.548317     0.337 2014-09-08 07:14:00           55
..         …         …       …                   …           …
968  55.214750  22.509733     0.191 2014-10-01 10:37:00           45
969  53.392167   7.292517     0.184 2014-10-03 11:28:00           40
970  53.107067   5.922067     0.181 2014-10-04 07:11:00           24
971  53.014967   5.811383     0.190 2014-10-04 14:12:00           21
```

```
972  53.009167   5.729433     0.193 2014-10-04 21:05:00           50

     Minimum_Distance  Average_Distance        Kp             N             E  \
0          327.950987        665.008368  1.308696   6949.105566  3851.158789
1          340.038476        667.146029  1.308696   6985.653894  3866.337781
2          348.223318        678.815409  1.190909   7035.551690  3877.443099
3          355.472899        680.040733  1.190909   7082.978312  3886.943680
4          355.980432        680.062802  1.190909   7086.091085  3888.485656
..                ...               ...       ...           ...           ...
968        240.371734        825.618960  2.406667  16968.785159  1828.024623
969        100.997905        755.121540  0.300000  18345.670851   502.789527
970        352.394408        784.016294  1.000000  18525.835109   337.275930
971        179.979519        889.678639  1.000000  18582.545311   321.253970
972        100.916767        883.585143  1.700000  18585.390848   318.935806

                C             H          D          I             F
0    57703.400422   7944.903535  28.994996  82.160510  58247.780322
1    57645.097979   7984.229967  28.963136  82.114324  58195.405739
2    57609.988412   8033.277823  28.860100  82.061723  58167.381903
3    57575.069804   8079.412908  28.756769  82.011941  58139.191393
4    57584.964457   8082.883617  28.755736  82.009909  58149.472389
..            ...           ...        ...        ...           ...
968  47875.310888  17066.966449   6.148685  70.379467  50826.437377
969  45904.683940  18352.559393   1.569880  68.208613  49437.399243
970  45661.727719  18528.905028   1.042995  67.913358  49277.923047
971  45603.873424  18585.322014   0.990427  67.827226  49245.583209
972  45607.600808  18588.127206   0.983131  67.825840  49250.093649

[973 rows x 15 columns]
```

The previous dataframe (GPS_ResInt), MagGeo has computed the geomagnetic components for each locations and time of your CSV trajectory. Now we will finish up combining the original atributes from your CSV with the annotated results from MagGeo.

[13]:
```
%%time
os.chdir(r"./data")
originalGPSTrack=pd.read_csv(gpsfilename)
MagGeoResult = pd.concat([originalGPSTrack, GPS_ResInt], axis=1)
#Drop duplicated columns. Latitude, Longitued, and DateTime will not be part of
↪the final result.
MagGeoResult.drop(columns=['Latitude', 'Longitude', 'DateTime'], inplace=True)
os.chdir(r"../")
MagGeoResult
```

```
CPU times: user 5.39 ms, sys: 2.29 ms, total: 7.67 ms
Wall time: 8.03 ms
```

```
[13]:            timestamp  location-long  location-lat  height  individual_id  \
0    08/09/2014 05:54      68.307333     70.854717   0.000              1
1    08/09/2014 06:10      67.975050     70.830300   0.406              1
2    08/09/2014 06:26      67.752417     70.761717   0.498              1
3    08/09/2014 06:42      67.561983     70.686517   0.787              1
4    08/09/2014 07:14      67.548317     70.685450   0.337              1
..                …              …            …     …              …
968  01/10/2014 10:37      22.509733     55.214750   0.191              6
969  03/10/2014 11:28       7.292517     53.392167   0.184              6
970  04/10/2014 07:11       5.922067     53.107067   0.181              6
971  04/10/2014 14:12       5.811383     53.014967   0.190              6
972  04/10/2014 21:05       5.729433     53.009167   0.193              6

     Altitude  TotalPoints  Minimum_Distance  Average_Distance        Kp  \
0       0.000           46        327.950987        665.008368  1.308696
1       0.406           46        340.038476        667.146029  1.308696
2       0.498           55        348.223318        678.815409  1.190909
3       0.787           55        355.472899        680.040733  1.190909
4       0.337           55        355.980432        680.062802  1.190909
..        …           …                …                …       …
968     0.191           45        240.371734        825.618960  2.406667
969     0.184           40        100.997905        755.121540  0.300000
970     0.181           24        352.394408        784.016294  1.000000
971     0.190           21        179.979519        889.678639  1.000000
972     0.193           50        100.916767        883.585143  1.700000

                N            E             C            H          D  \
0     6949.105566  3851.158789  57703.400422   7944.903535  28.994996
1     6985.653894  3866.337781  57645.097979   7984.229967  28.963136
2     7035.551690  3877.443099  57609.988412   8033.277823  28.860100
3     7082.978312  3886.943680  57575.069804   8079.412908  28.756769
4     7086.091085  3888.485656  57584.964457   8082.883617  28.755736
..            …           …            …            …         …
968  16968.785159  1828.024623  47875.310888  17066.966449   6.148685
969  18345.670851   502.789527  45904.683940  18352.559393   1.569880
970  18525.835109   337.275930  45661.727719  18528.905028   1.042995
971  18582.545311   321.253970  45603.873424  18585.322014   0.990427
972  18585.390848   318.935806  45607.600808  18588.127206   0.983131

             I             F
0    82.160510  58247.780322
1    82.114324  58195.405739
2    82.061723  58167.381903
3    82.011941  58139.191393
4    82.009909  58149.472389
..         …            …
968  70.379467  50826.437377
```

```
969  68.208613  49437.399243
970  67.913358  49277.923047
971  67.827226  49245.583209
972  67.825840  49250.093649

[973 rows x 17 columns]
```

1.11 Export the final results to a CSV file

[14]:
```
%%time
#Exporting the CSV file
os.chdir(r"./results")
outputfile ="GeoMagResult_"+gpsfilename
export_csv = MagGeoResult.to_csv (outputfile, index = None, header=True)
os.chdir(r"../")
```

```
CPU times: user 18 ms, sys: 1.79 ms, total: 19.8 ms
Wall time: 25.6 ms
```

1.12 Validate the results (Optional)

To validate the results we plot the Fgpscolumn.

[15]:
```
## Creating a copy of the results and setting the Datetime Column as dataframe␣
↪index.
ValidateDF = GPS_ResInt.copy()
ValidateDF.set_index("DateTime", inplace=True)
## Plotting the F column.
hist = ValidateDF.hist(column='F')
plt.title('F distribution')
plt.xlabel('F in nT')
plt.ylabel('# of measurements')
```

[15]:
```
Text(0, 0.5, '# of measurements')
```

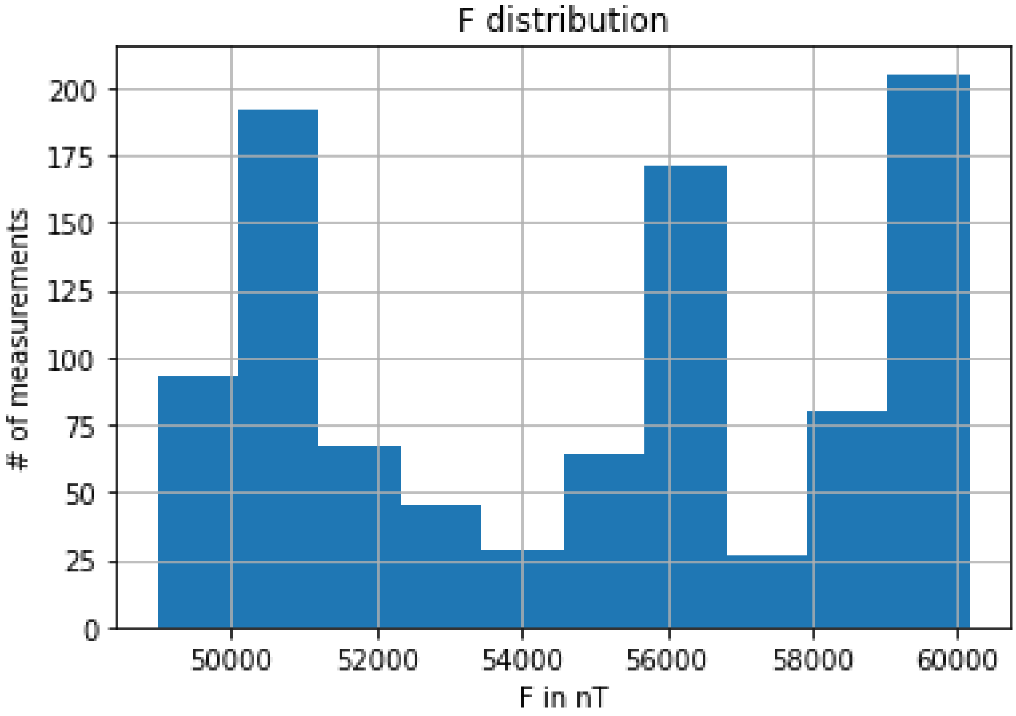

1.13 Map the GPS Track using the annotated Magnetic Values (Optional)

Now we are going to plot the annotated GPS track stored into the MagDataFinal dataframe to see the different magnetic components in a map to have a better prespective of the impact of the earth magnetic field.

[16]:
```
ValidateDF.plot(kind="scatter", x="Latitude", y="Longitude",
    label="Magnetic Intensity in nT",
    c="F", cmap=plt.get_cmap("gist_rainbow"),
    colorbar=True, alpha=0.4, figsize=(10,7),
    sharex=False #This is only needed to get the x-axis label working due to a␣
 ↪current bug in pandas plot.

)

plt.ylabel("Longitude", fontsize=12)
plt.xlabel("Latitude", fontsize=12)
plt.legend(fontsize=12)
plt.show()
```

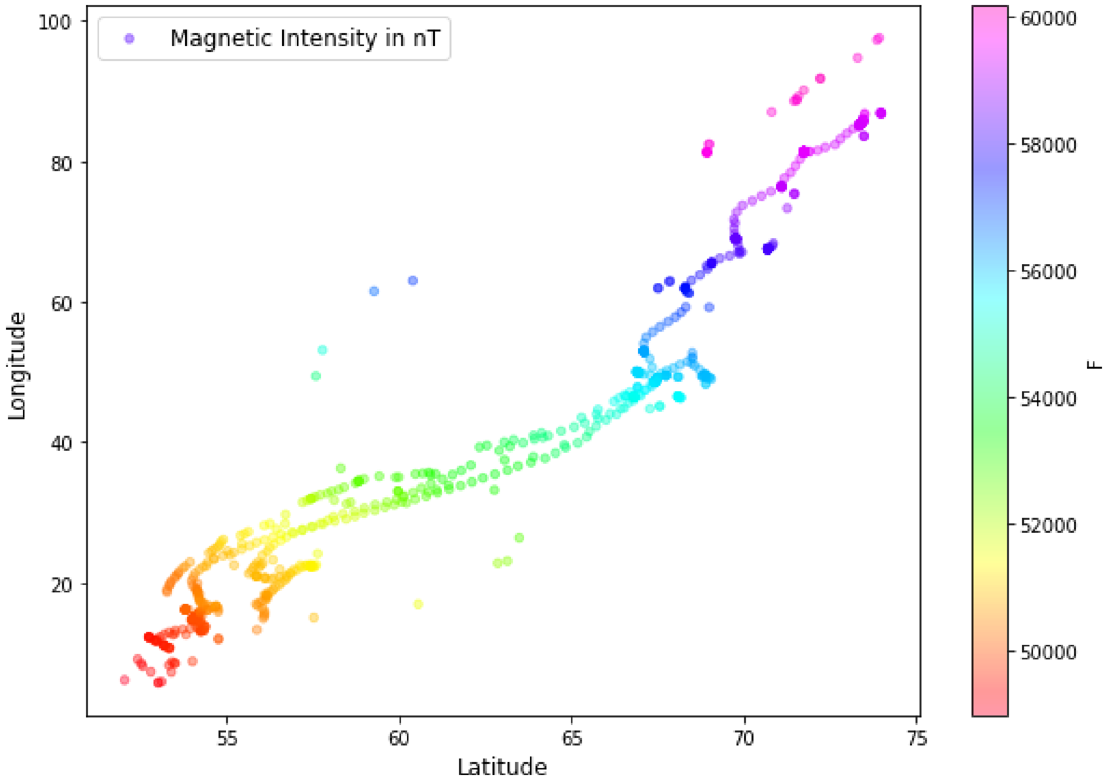

[17]:
```python
import geopandas
import geoplot
gdf = geopandas.GeoDataFrame(ValidateDF, geometry=geopandas.
 ↪points_from_xy(ValidateDF.Longitude, ValidateDF.Latitude))
gdf.head()
```

[17]:
```
                      Latitude  Longitude  Altitude  TotalPoints  \
DateTime
2014-09-08 05:54:00  70.854717  68.307333     0.000           46
2014-09-08 06:10:00  70.830300  67.975050     0.406           46
2014-09-08 06:26:00  70.761717  67.752417     0.498           55
2014-09-08 06:42:00  70.686517  67.561983     0.787           55
2014-09-08 07:14:00  70.685450  67.548317     0.337           55

                     Minimum_Distance  Average_Distance        Kp  \
DateTime
2014-09-08 05:54:00        327.950987        665.008368  1.308696
2014-09-08 06:10:00        340.038476        667.146029  1.308696
2014-09-08 06:26:00        348.223318        678.815409  1.190909
2014-09-08 06:42:00        355.472899        680.040733  1.190909
2014-09-08 07:14:00        355.980432        680.062802  1.190909
```

```
                             N            E             C            H  \
DateTime
2014-09-08 05:54:00  6949.105566  3851.158789  57703.400422  7944.903535
2014-09-08 06:10:00  6985.653894  3866.337781  57645.097979  7984.229967
2014-09-08 06:26:00  7035.551690  3877.443099  57609.988412  8033.277823
2014-09-08 06:42:00  7082.978312  3886.943680  57575.069804  8079.412908
2014-09-08 07:14:00  7086.091085  3888.485656  57584.964457  8082.883617

                             D          I             F  \
DateTime
2014-09-08 05:54:00  28.994996  82.160510  58247.780322
2014-09-08 06:10:00  28.963136  82.114324  58195.405739
2014-09-08 06:26:00  28.860100  82.061723  58167.381903
2014-09-08 06:42:00  28.756769  82.011941  58139.191393
2014-09-08 07:14:00  28.755736  82.009909  58149.472389

                                     geometry
DateTime
2014-09-08 05:54:00  POINT (68.30733 70.85472)
2014-09-08 06:10:00  POINT (67.97505 70.83030)
2014-09-08 06:26:00  POINT (67.75242 70.76172)
2014-09-08 06:42:00  POINT (67.56198 70.68652)
2014-09-08 07:14:00  POINT (67.54832 70.68545)
```

[21]:
```
world = geopandas.read_file(geopandas.datasets.get_path('naturalearth_lowres'))

ax = world.plot(color='white', edgecolor='gray', figsize = (18,8))

minx, miny, maxx, maxy = gdf.total_bounds
ax.set_xlim(minx, maxx)
ax.set_ylim(miny, maxy)

gdf.plot(ax=ax, column='F', legend=True,
         legend_kwds={'label': "Magnetic Intensity in nT",
                      'orientation': "horizontal"})
plt.ylabel("Longitude", fontsize=12)
plt.xlabel("Latitude", fontsize=12)

plt.show()
```

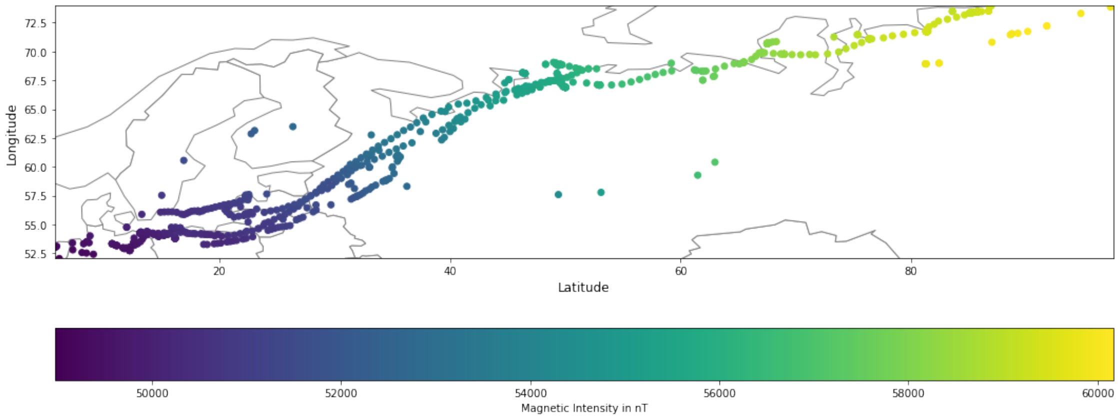

[19]:
```
fig, (ax1, ax2) = plt.subplots(ncols=2, figsize = (18,8))

ax1 = world.plot(ax=ax1, color='white', edgecolor='black')
xlim = ([gdf.total_bounds[0],  gdf.total_bounds[2]])
ylim = ([gdf.total_bounds[1],  gdf.total_bounds[3]])
ax1.set_xlim(xlim)
ax1.set_ylim(ylim)


gdf.plot(ax=ax1, column='F', legend=True,
         legend_kwds={'label': "Magnetic Intensity in nT",
                      'orientation': "horizontal"})
plt.ylabel("Longitude", fontsize=9)
plt.xlabel("Latitude", fontsize=9)
ax1.set_title('Magnetic Intensity - F')
ax1.set_xlabel('Latitude')
ax1.set_ylabel('Longitude')


ax2 = world.plot( ax=ax2, color='white', edgecolor='black')
xlim = ([gdf.total_bounds[0],  gdf.total_bounds[2]])
ylim = ([gdf.total_bounds[1],  gdf.total_bounds[3]])
ax2.set_xlim(xlim)
ax2.set_ylim(ylim)


# We can now plot our ``GeoDataFrame``.
gdf.plot(ax=ax2, column='I', legend=True, cmap='Spectral',
         legend_kwds={'label': " Inclination in Degrees",
                      'orientation': "horizontal"})
ax2.set_title('Inclination - I')
ax2.set_xlabel('Latitude')
```

```
ax2.set_ylabel('Longitude')
```

[19]: Text(686.1045454545454, 0.5, 'Longitude')

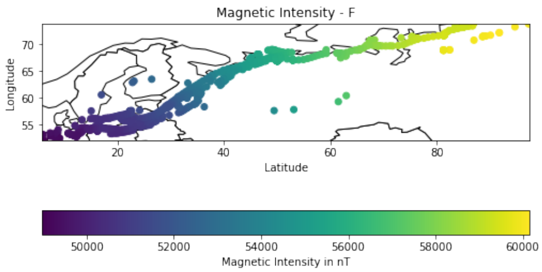

MagGeo - Parallel Mode

April 26, 2021

1 MagGeo - Parallel Mode

Authors | Fernando Benitez-Paez, Urška Demšar, Jed Long, Ciaran Beggan

Contact | Fernando.Benitez@st-andrews.ac.uk, ud2@st-andrews.ac.uk, jed.long@uwo.ca, ciar@bgs.ac.uk

Keywords | Bird migration, data fusion, Earth's magnetic field, Swarm, GPS tracking

1.1 Overview

This Jupyter Notebook will guide you through the required steps to annotate your GPS tracking data with the earth's magnetic field data from Swarm (European Space Agency). This version is called Parallel Mode to take advantage of parallelized computing to process big datasets.

To execute the code, you can go through each cell (pressing Crtl+Enter), you will also find inner comments `##` to describe each particular step. If you are not familiar with Jupyter Notebook, you migth want to take some time to learn how to use it first, for example take a look at the notebook-basics.ipynb Notebook inside MagGeo.

For parallel processing, there are some considerations to make:

1. Linux and Windows environments have some differences. In windows we need to separate the functions and store them separately, then import them into a `main` function.
2. Defining what part of the process is CPU bound and what part is I/O bound: Identify what parts of the program are I/O bound (writing or reading from the disk or network) and what part par CPU bound (Processing capacity). To take advantage of our CPU capacity we need to identify the process where the CPU is actually doing the main Tasks.

1.2 Data requirements

Your trajectory must be in a csv format:

There are three columns that must be included in your GPS trajectory. Make sure your GPS trajectory includes **Latitude** , **Longitude** and **timestamp**. We suggest that the Timestamp column follow the day/month/year Hour:Minute (**dd/mm/yyyy HH:MM:SS**) format, Latitude and Longitude should be in decimal degrees (WGS84). Optionally an altitude column can be used providing altitude (the altitude must be in **km**). Other Columns will be ignored. Here it is an example of how your GPS track should look:

For this example we are reading the BirdGPSTrajectory.csv file. If you want to run the method using your own csv file, make sure you store your the file in the ./data folder. For more information

about the dataset we used in this example go to the Main Notebook.

1.3 Import the requeried libraries

[1]:
```
import datetime as dt
import sys,os
from pathlib import Path
import pandas as pd
import numpy as np
import datetime, time
from datetime import timedelta
import math
import pathlib
from datetime import datetime
import time
import calendar
import datetime
from viresclient import ClientConfig
import matplotlib.pyplot as plt

from viresclient import set_token
from MagGeoFunctions import getGPSData
from MagGeoFunctions import Get_Swarm_residuals
```

1.4 Add your VirES web client Token

The **VirES client API**, requires a token. Before start you need to get your own VirES token. You can visit https://vires.services/ to get yours, and then add it into the next cell.

[2]:
```
set_token("https://vires.services/ows", set_default=True)
```

```
Enter token:········
Token saved for https://vires.services/ows
```

1.5 Reading the GPS track

The following steps will load the GPS track from a csv file, and set some requirements before download the data from Swarm. Importing the GPS track. You can note that there is a folder to store the CSV file. Using `os.getcwd()` you can validate where the file is located.

[3]:
```
#Make sure the csv file of your trackectory is stored in the Data folder.
#Enter the name of your GPS track csv file including the extension .csv  and␣
 ↪press Enter (e.g. BirdGPSTrajectory.csv)
os.chdir(r"./data")
gpsfilename=input("What is the name of your .csv file?: ") # i.e␣
 ↪BirdGPSTrajectory.csv
Lat=input("Enter the name of your Latitude column?: ") #i.e location-lat
Long=input("Enter the name of your Longitud column?: ") # i.e location-long
```

```
DateTime=input("Enter the date and time column name?: ") # i.e timestamp
altitude = input("Enter the Altitude column name?, if you don't have the␣
 ↪altitude column, just press Enter: ")
# i.e height (Only in KM)
#If your csv track file doesnt not have any altitude attribute, MagGeo will use␣
 ↪sea level as your altitude (i.e. 0 Km).
```

```
What is the name of your .csv file?: BirdGPSTrajectory.csv
Enter the name of your Latitude column?: location-lat
Enter the name of your Longitud column?: location-long
Enter the date and time column name?: timestamp
Enter the Altitude column name?, if you don't have the altitude column, just
press Enter: height
```

[4]:
```
# Here MagGeo is reading your CSV file, taking the Lat, Long, Date&Time and␣
 ↪Altitutes attributes and compute, some aditional attrubutes we need to the␣
 ↪annotation process.
# Setting the date and time attributes for the required format and computing␣
 ↪the epoch column. Values like Maximum and Minimun Date and time are also␣
 ↪calculated.
GPSData = getGPSData(gpsfilename,Lat,Long,DateTime,altitude)
os.chdir(r"../")
GPSData
```

[4]:
```
             gpsDateTime    gpsLong     gpsLat  gpsAltitude       epoch  \
0    2014-09-08 05:54:00  68.307333  70.854717        0.000  1410155640
1    2014-09-08 06:10:00  67.975050  70.830300        0.406  1410156600
2    2014-09-08 06:26:00  67.752417  70.761717        0.498  1410157560
3    2014-09-08 06:42:00  67.561983  70.686517        0.787  1410158520
4    2014-09-08 07:14:00  67.548317  70.685450        0.337  1410160440
..                   …          …          …           …           …
968  2014-10-01 10:37:00  22.509733  55.214750        0.191  1412159820
969  2014-10-03 11:28:00   7.292517  53.392167        0.184  1412335680
970  2014-10-04 07:11:00   5.922067  53.107067        0.181  1412406660
971  2014-10-04 14:12:00   5.811383  53.014967        0.190  1412431920
972  2014-10-04 21:05:00   5.729433  53.009167        0.193  1412456700

          dates     times
0    2014-09-08  05:54:00
1    2014-09-08  06:10:00
2    2014-09-08  06:26:00
3    2014-09-08  06:42:00
4    2014-09-08  07:14:00
..          …         …
968  2014-10-01  10:37:00
969  2014-10-03  11:28:00
970  2014-10-04  07:11:00
```

```
971  2014-10-04  14:12:00
972  2014-10-04  21:05:00

[973 rows x 7 columns]
```

Setting the date and time attributes for the requerided format and computing the epoch column. Values like Maximum and Minimun Date and time are also calculated.

1.6 Validate the right amount of Swarm measures

The following loop is identifiying the time and validating if the time is less than 4:00 hours and more than 20:00 hours to bring one extra day of data. The result of this validation is written in a empty python list which will be later validated to get the unique dates avoing to download data for the same day and reducing the the downloand time process.

[5]:
```
%%time
datestimeslist = []
for index, row in GPSData.iterrows():
    datetimerow  = row['gpsDateTime']
    daterow = row['dates']
    hourrow = row['times']
    hourrow = hourrow.strftime('%H:%M:%S')
    if hourrow < '04:00:00':
        date_bfr = daterow - (timedelta(days=1))
        datestimeslist.append(daterow)
        datestimeslist.append(date_bfr)
    if hourrow > '20:00:00':
        Date_aft = daterow + (timedelta(days=1))
        datestimeslist.append(daterow)
        datestimeslist.append(Date_aft)
    else:
        datestimeslist.append(daterow)
```

```
CPU times: user 90.1 ms, sys: 1.9 ms, total: 92 ms
Wall time: 90.8 ms
```

Getting a list of unique dates, to being used to download the Swarm Data

[6]:
```
%%time
def uniquelistdates(list):
    x = np.array(list)
    uniquelist = np.unique(x)
    return uniquelist

uniquelist_dates = uniquelistdates(datestimeslist)
uniquelist_dates
```

```
CPU times: user 2.18 ms, sys: 39 µs, total: 2.22 ms
Wall time: 2.21 ms
```

[6]:
```
array([datetime.date(2014, 8, 18), datetime.date(2014, 8, 19),
       datetime.date(2014, 8, 25), datetime.date(2014, 8, 26),
       datetime.date(2014, 8, 27), datetime.date(2014, 8, 28),
       datetime.date(2014, 8, 29), datetime.date(2014, 8, 30),
       datetime.date(2014, 8, 31), datetime.date(2014, 9, 3),
       datetime.date(2014, 9, 5), datetime.date(2014, 9, 6),
       datetime.date(2014, 9, 8), datetime.date(2014, 9, 13),
       datetime.date(2014, 9, 14), datetime.date(2014, 9, 15),
       datetime.date(2014, 9, 17), datetime.date(2014, 9, 18),
       datetime.date(2014, 9, 19), datetime.date(2014, 9, 20),
       datetime.date(2014, 9, 21), datetime.date(2014, 9, 22),
       datetime.date(2014, 9, 23), datetime.date(2014, 9, 24),
       datetime.date(2014, 9, 25), datetime.date(2014, 9, 26),
       datetime.date(2014, 9, 27), datetime.date(2014, 9, 29),
       datetime.date(2014, 9, 30), datetime.date(2014, 10, 1),
       datetime.date(2014, 10, 2), datetime.date(2014, 10, 3),
       datetime.date(2014, 10, 4), datetime.date(2014, 10, 5),
       datetime.date(2014, 10, 6), datetime.date(2014, 10, 10),
       datetime.date(2014, 10, 13), datetime.date(2014, 10, 14),
       datetime.date(2014, 10, 15), datetime.date(2014, 10, 22),
       datetime.date(2014, 10, 23), datetime.date(2014, 10, 24)],
      dtype=object)
```

1.7 Download Swarm residuals data

Once the date and time columns have been defined, and the unique dates were identified the script can start the download process. Usually the data from Swarm is requested using only one satellite, however **MagGeo** will use the magnetic measures from the three satellite of the Swarm Mission.

Be aware: Due to the amount of dates the GPS track has (42 days) to request and compute the residuals, the time to process the sample data will take approximately 10 minutes.

Set a connection to the VirES client and using the function Get_Swarm_residuals we will get the swarm residuals for the dates included in the previous list.

[ ]:
```
%%time

hours_t_day = 24
hours_added = datetime.timedelta(hours = hours_t_day)

listdfa = []
listdfb = []
listdfc = []

for d in uniquelist_dates:
    print("Getting Swarm data for date:",d )
    startdate = datetime.datetime.combine(d, datetime.datetime.min.time())
    enddate = startdate + hours_added
```

```
   SwarmResidualsA,SwarmResidualsB,SwarmResidualsC =␣
↪Get_Swarm_residuals(startdate, enddate)
   listdfa.append(SwarmResidualsA)
   listdfb.append(SwarmResidualsB)
   listdfc.append(SwarmResidualsC)
```

Concat the previous results and temporally save the requested data locally: Integrate the previous list for all dates, into pandas dataframes. We will temporally saved the previous results, in case you need to re-run MagGeo, with the following csv files you will not need to run the download process.

[8]:
```
%%time
os.chdir(r"./temp_data")
TotalSwarmRes_A = pd.concat(listdfa, join='outer', axis=0)
TotalSwarmRes_A.to_csv ('TotalSwarmRes_A.csv', header=True)
TotalSwarmRes_B = pd.concat(listdfb, join='outer', axis=0)
TotalSwarmRes_B.to_csv ('TotalSwarmRes_B.csv', header=True)
TotalSwarmRes_C = pd.concat(listdfc, join='outer', axis=0)
TotalSwarmRes_C.to_csv ('TotalSwarmRes_C.csv', header=True)
os.chdir(r"../")
TotalSwarmRes_A #If you need to take a look of the Swarm Data, you can print␣
↪TotalSwarmRes_B, or TotalSwarmRes_C
```

```
CPU times: user 3.99 s, sys: 173 ms, total: 4.17 s
Wall time: 4.19 s
```

[8]:
```
             Kp   Longitude      Radius   Latitude     F_res  Flags_B  \
epoch
1408320000  1.0 -134.672531  6832723.53  66.843847  6.172334        0
1408320030  0.7 -134.175850  6832542.55  68.750719  5.200886        0
1408320060  0.7 -133.562740  6832377.42  70.654918  2.146256        0
1408320090  0.7 -132.795266  6832228.58  72.555551 -2.443055        0
1408320120  0.7 -131.817227  6832096.43  74.451293 -7.002322        0
…           …         …           …          …         …        …
1414195050  1.7  126.692648  6846883.18 -31.747197  3.577492        0
1414195080  1.7  126.687015  6846698.22 -29.835345  5.393771        0
1414195110  1.7  126.676892  6846503.98 -27.923204  6.468472        0
1414195140  1.7  126.662769  6846300.35 -26.010783  6.738029        0
1414195170  1.7  126.645088  6846087.25 -24.098091  6.425266        0

            Flags_F Spacecraft      N_res      E_res     C_res  \
epoch
1408320000        1          A  -2.784954   9.087620  6.157086
1408320030        1          A -16.267735  24.676547  6.178650
1408320060        1          A -29.700036  34.292158  4.071577
1408320090        1          A -37.796721  44.267360 -0.604402
1408320120        1          A -72.888506  62.526620 -3.285812
```

```
…                   …        …        …        …        …
1414195050          1        A  -9.562833   6.730607 -8.331892
1414195080          1        A  -7.087156   5.130584 -9.583105
1414195110          1        A  -4.444862   4.759146 -9.769941
1414195140          1        A  -2.557345   1.961955 -9.392708
1414195170          1        A  -1.458364   0.887033 -8.652785

                     timestamp
epoch
1408320000 2014-08-18 00:00:00
1408320030 2014-08-18 00:00:30
1408320060 2014-08-18 00:01:00
1408320090 2014-08-18 00:01:30
1408320120 2014-08-18 00:02:00
…                          …
1414195050 2014-10-24 23:57:30
1414195080 2014-10-24 23:58:00
1414195110 2014-10-24 23:58:30
1414195140 2014-10-24 23:59:00
1414195170 2014-10-24 23:59:30

[120960 rows x 12 columns]
```

1.8 Set the number of processes, and split the dataframe (GPSData) into chunks

We can set the number or processess we need to dedicate for the multiprocessing mode, of course that also depends on the number of cores the machine you are using to run **MagGeo**. You can use `multiprocessing.cpu_count()` to set the number of processes as the the number of cores your machine has. Beside that we will also to split the GPS track into chucks to dedicate each core for each chuck. For more information take a look at the Home Notebook.

[ ]:
```
import multiprocessing
import sklearn
from multiprocessing import Pool

NumCores = multiprocessing.cpu_count()
df_chunks = np.array_split(GPSData,NumCores)
df_chunks
```

1.9 Spatio-Temporal filter and Interpolation process (ST-IDW)

Once we have requested the swarm data, now we need to `filter` in space and time the available points to compute the magnetic values (NEC frame) for each GPS point based on its particular date and time. The function ST_IDW_Process imported in the row_handler, takes the GPS track and the downloaded data from swarm to filter in space and time based on the criteria defined in our method. With the swarm data filtered we interpolated (IDW) the NEC components for each GPS data point, based on the latitude, date, time and number of Swarm points filtered.

The function CHAOS_ground_values, inside the MagGeoFunctions file, is used to run the **Calculation of magnetic components**. This calculation requeries the magnetic components at the trajectory altitude (or at the ground level) using CHAOS (theta, phi, radial). This process include a rotation and transformation between a geocentric frame (CHAOS) and geodetic frame (GPS track). Once the corrected values are calculated, are included in the GPS track, and the non-necesary columns are removed. For more information about this process go to the Main Notebook.

1.9.1 Run the (ST-IDW) process in parallel mode

Although the next cell seems to run a small `main` function. What is happening is a call for several functions running at same time for several cores. Initially we set a pool of processes. Using the `pool` class we will distribute the assigned function among the data chucks we created. Every data chunk will be like a subset of the entire GPS track. So we need to iterate among data chunk. And inside every data chunk we need to identify the `datetime`, `epoch`, `altitude`, `latitude` and `longitude` of each row to run the interpolation & annotation process using the Swarm data we have filtered and stored in the previous steps.

The function in charge to distribute the required function (`row_handler`) among the data chunks is the map function from the `pool` class.

`row_handler.py` is an interows iteration to get the required parameter for the `ST_IDW_Process` function.

Auxiliary Functions:

ST_IDW_Process function: This is the main function in charge to read the Swarm Data already filtered, and then import DfTime_func, distance_to_GPS, Kradius , DistJ functions to compute the spatial-time cylinder and the annotation process. The return of this function is a row (dictionary) that will be appended into a python list where all the results from the different cores. The python list from every process is concatenated into a pandas dataframe in the main function having there the whole chain of the parallel process.

distance_to_GPS function: Is the function in charge to calculate the distance between each GPS Point and the Swarm Point.

Kradius function: Is the function in charge to compute the R (radius) value in the cylinder. The R value will be considered based on the latitude of each GPS Point.

DistJ function: This function will calculate the d value as the hypotenuse created in the triangle created amount the locations of the GPS point, the location of the Swarm points and the radius value.

DfTime_func function: This is a time function to selected the points in the range of a the DeltaTime - DT window. The Delta time window has been set as 4 hours for each satellite trajectory.

CHAOS_ground_values function: This is the calculation of geomagnetic components function to get the CHAOS magnetic values and process the Nres,Eres,Cres values and transform them into the N,E,C values at the GPS altitude.

[10]:
```
%%time
from functools import partial
import row_handler
```

```
if __name__ == '__main__':
    with multiprocessing.Pool(NumCores) as pool:
        GeoMagParallelResult = pd.concat(pool.map(partial(row_handler.
 ↪row_handler), df_chunks), ignore_index=True)
```

```
CPU times: user 846 ms, sys: 399 ms, total: 1.25 s
Wall time: 26.3 s
```

With the Parallel mode the Annotation process takes about 12 seconds to complete (We had tested the parallel process in a windows server machine with 12 cores, see the image bellow). With the same GPS track in the sequetial mode the process is complete in about 2 minutes. In the image bellow you can see how the machine create several python processes and all cores (full CPU capacity) is taken.

Multiprocessing:

is even more powerfull when you have to process a big amount of data (e.g. 2 millons of points). Although here is making a notable improvement if you have to process a big dataset the parallelization makes even more sense.

Be aware that there is no output cell in here, you can follow the parallelization progress in the Anaconda Prompt.

1.10 The final result

With the NEC components for each GPS Track point, it is possible to compute the aditional magnetic components. For more information about the magnetic components and their relevance go to the main paper or notebook.

[11]:
```
#14. Having Intepolated and weigth magnetic values, we can compute the other␣
 ↪magnectic components.
GeoMagParallelResult['H'] = np.
 ↪sqrt((GeoMagParallelResult['N']**2)+(GeoMagParallelResult['E']**2))
#check the arcgtan in python., From arctan2 is saver.
DgpsRad = np.arctan2(GeoMagParallelResult['E'],GeoMagParallelResult['N'])
GeoMagParallelResult['D'] = np.degrees(DgpsRad)
IgpsRad = np.arctan2(GeoMagParallelResult['C'],GeoMagParallelResult['H'])
GeoMagParallelResult['I'] = np.degrees(IgpsRad)
GeoMagParallelResult['F'] = np.sqrt((GeoMagParallelResult['N']**2)+
                                    (GeoMagParallelResult['E']**2)+
                                    (GeoMagParallelResult['C']**2))
GeoMagParallelResult
```

[11]:
```
    Latitude  Longitude  Altitude            DateTime  TotalPoints  \
0  70.854717  68.307333     0.000 2014-09-08 05:54:00           46
1  70.830300  67.975050     0.406 2014-09-08 06:10:00           46
2  70.761717  67.752417     0.498 2014-09-08 06:26:00           55
3  70.686517  67.561983     0.787 2014-09-08 06:42:00           55
```

```
4    70.685450  67.548317     0.337 2014-09-08 07:14:00           55
..         …          …       …                   …          …
968  55.214750  22.509733     0.191 2014-10-01 10:37:00           45
969  53.392167   7.292517     0.184 2014-10-03 11:28:00           40
970  53.107067   5.922067     0.181 2014-10-04 07:11:00           24
971  53.014967   5.811383     0.190 2014-10-04 14:12:00           21
972  53.009167   5.729433     0.193 2014-10-04 21:05:00           50

     Minimum_Distance  Average_Distance        Kp             N            E  \
0          327.950987        665.008368  1.308696   6949.105566  3851.158789
1          340.038476        667.146029  1.308696   6985.653894  3866.337781
2          348.223318        678.815409  1.190909   7035.551690  3877.443099
3          355.472899        680.040733  1.190909   7082.978312  3886.943680
4          355.980432        680.062802  1.190909   7086.091085  3888.485656
..                …                 …       …             …            …
968        240.371734        825.618960  2.406667  16968.785159  1828.024623
969        100.997905        755.121540  0.300000  18345.670851   502.789527
970        352.394408        784.016294  1.000000  18525.835109   337.275930
971        179.979519        889.678639  1.000000  18582.545311   321.253970
972        100.916767        883.585143  1.700000  18585.390848   318.935806

                C             H          D          I             F
0    57703.400422   7944.903535  28.994996  82.160510  58247.780322
1    57645.097979   7984.229967  28.963136  82.114324  58195.405739
2    57609.988412   8033.277823  28.860100  82.061723  58167.381903
3    57575.069804   8079.412908  28.756769  82.011941  58139.191393
4    57584.964457   8082.883617  28.755736  82.009909  58149.472389
..            …             …          …          …             …
968  47875.310888  17066.966449   6.148685  70.379467  50826.437377
969  45904.683940  18352.559393   1.569880  68.208613  49437.399243
970  45661.727719  18528.905028   1.042995  67.913358  49277.923047
971  45603.873424  18585.322014   0.990427  67.827226  49245.583209
972  45607.600808  18588.127206   0.983131  67.825840  49250.093649

[973 rows x 15 columns]
```

The previous dataframe (GPS_ResInt), MagGeo has computed the geomagnetic components for each locations and time of your CSV trajectory. Now we will finish up combining the original atributes from your CSV with the annotated results from MagGeo.

[12]:
```
%%time
os.chdir(r"./data")
originalGPSTrack=pd.read_csv(gpsfilename)
MagGeoResult = pd.concat([originalGPSTrack, GeoMagParallelResult], axis=1)
#Drop duplicated columns. Latitude, Longitued, and DateTime will not be part of␣
 ↪the final result.
# MagGeoResult.drop(columns=['Latitude', 'Longitude', 'DateTime'], inplace=True)
```

```
os.chdir(r"../")
MagGeoResult
```

```
CPU times: user 5.62 ms, sys: 2.69 ms, total: 8.31 ms
Wall time: 8.04 ms
```

[12]:

	timestamp	location-long	location-lat	height	individual_id	\
0	08/09/2014 05:54	68.307333	70.854717	0.000	1	
1	08/09/2014 06:10	67.975050	70.830300	0.406	1	
2	08/09/2014 06:26	67.752417	70.761717	0.498	1	
3	08/09/2014 06:42	67.561983	70.686517	0.787	1	
4	08/09/2014 07:14	67.548317	70.685450	0.337	1	
..	…	…	…	…	…	
968	01/10/2014 10:37	22.509733	55.214750	0.191	6	
969	03/10/2014 11:28	7.292517	53.392167	0.184	6	
970	04/10/2014 07:11	5.922067	53.107067	0.181	6	
971	04/10/2014 14:12	5.811383	53.014967	0.190	6	
972	04/10/2014 21:05	5.729433	53.009167	0.193	6	

	Latitude	Longitude	Altitude	DateTime	TotalPoints	\
0	70.854717	68.307333	0.000	2014-09-08 05:54:00	46	
1	70.830300	67.975050	0.406	2014-09-08 06:10:00	46	
2	70.761717	67.752417	0.498	2014-09-08 06:26:00	55	
3	70.686517	67.561983	0.787	2014-09-08 06:42:00	55	
4	70.685450	67.548317	0.337	2014-09-08 07:14:00	55	
..	…	…	…	…	…	
968	55.214750	22.509733	0.191	2014-10-01 10:37:00	45	
969	53.392167	7.292517	0.184	2014-10-03 11:28:00	40	
970	53.107067	5.922067	0.181	2014-10-04 07:11:00	24	
971	53.014967	5.811383	0.190	2014-10-04 14:12:00	21	
972	53.009167	5.729433	0.193	2014-10-04 21:05:00	50	

	Minimum_Distance	Average_Distance	Kp	N	E	\
0	327.950987	665.008368	1.308696	6949.105566	3851.158789	
1	340.038476	667.146029	1.308696	6985.653894	3866.337781	
2	348.223318	678.815409	1.190909	7035.551690	3877.443099	
3	355.472899	680.040733	1.190909	7082.978312	3886.943680	
4	355.980432	680.062802	1.190909	7086.091085	3888.485656	
..	…	…	…	…	…	
968	240.371734	825.618960	2.406667	16968.785159	1828.024623	
969	100.997905	755.121540	0.300000	18345.670851	502.789527	
970	352.394408	784.016294	1.000000	18525.835109	337.275930	
971	179.979519	889.678639	1.000000	18582.545311	321.253970	
972	100.916767	883.585143	1.700000	18585.390848	318.935806	

	C	H	D	I	F
0	57703.400422	7944.903535	28.994996	82.160510	58247.780322

```
1    57645.097979   7984.229967  28.963136  82.114324  58195.405739
2    57609.988412   8033.277823  28.860100  82.061723  58167.381903
3    57575.069804   8079.412908  28.756769  82.011941  58139.191393
4    57584.964457   8082.883617  28.755736  82.009909  58149.472389
..            …             …          …          …            …
968  47875.310888  17066.966449   6.148685  70.379467  50826.437377
969  45904.683940  18352.559393   1.569880  68.208613  49437.399243
970  45661.727719  18528.905028   1.042995  67.913358  49277.923047
971  45603.873424  18585.322014   0.990427  67.827226  49245.583209
972  45607.600808  18588.127206   0.983131  67.825840  49250.093649

[973 rows x 20 columns]
```

1.11 Export the final results to a CSV file

[13]:
```
%%time
#Exporting the CSV file
os.chdir(r"./results")
outputfile ="GeoMagResult_"+gpsfilename
export_csv = MagGeoResult.to_csv (outputfile, index = None, header=True)
os.chdir(r"../")
```

```
CPU times: user 22.2 ms, sys: 2.8 ms, total: 25 ms
Wall time: 24.5 ms
```

1.12 Validate the results (optional)

To validate the results we plot the Fcolumn.

[14]:
```
## Creating a copy of the results and setting the Datetime Column as dataframe␣
↪index.
ValidateDF = GeoMagParallelResult.copy()
ValidateDF.set_index("DateTime", inplace=True)
## Plotting the F column.
hist = ValidateDF.hist(column='F')
plt.title('F distribution')
plt.xlabel('F in nT')
plt.ylabel('# of measurements')
```

[14]:
```
Text(0, 0.5, '# of measurements')
```

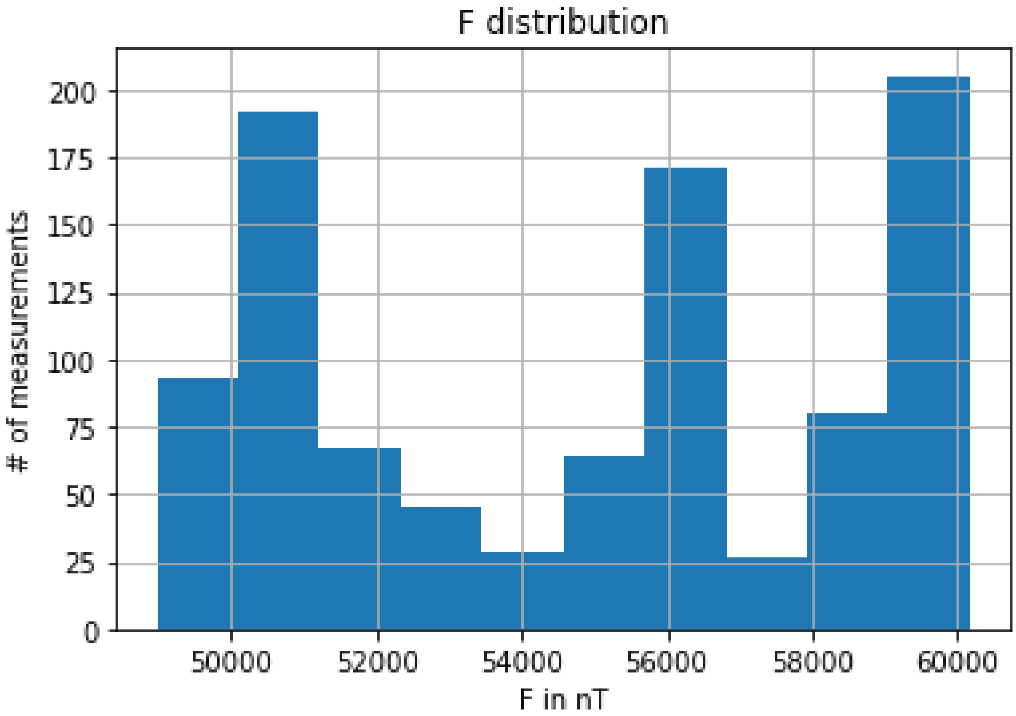

1.13 Map the GPS Track using the annotated Magnetic Values (optional)

Now we are going to plot the annotated GPS track stored into the MagDataFinal dataframe to see how the different magnetic components in a map to have a better prespective of the impact of the earth magnetic field.

[15]:
```
import matplotlib.pyplot as plt

ValidateDF.plot(kind="scatter", x="Latitude", y="Longitude",
    label="Magnetic Intensity in nT",
    c="F", cmap=plt.get_cmap("gist_rainbow"),
    colorbar=True, alpha=0.4, figsize=(10,7),
    sharex=False #This is only needed to get the x-axis label working due to a␣
↪current bug in pandas plot.
)

plt.ylabel("Longitude", fontsize=12)
plt.xlabel("Latitude", fontsize=10)
plt.legend(fontsize=12)
plt.show()
```

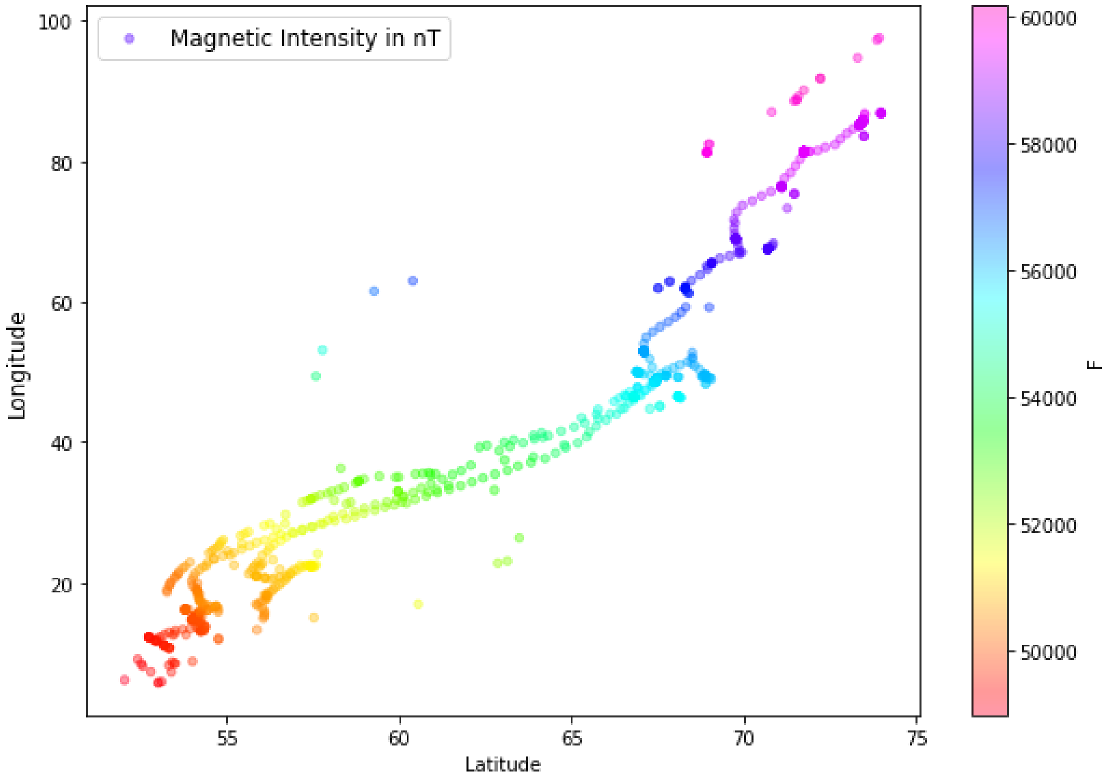

[16]:

```
import geopandas
import geoplot
gdf = geopandas.GeoDataFrame(ValidateDF, geometry=geopandas.
 ↪points_from_xy(ValidateDF.Longitude, ValidateDF.Latitude))
gdf.head()
```

[16]:

```
                      Latitude  Longitude  Altitude  TotalPoints  \
DateTime
2014-09-08 05:54:00  70.854717  68.307333     0.000           46
2014-09-08 06:10:00  70.830300  67.975050     0.406           46
2014-09-08 06:26:00  70.761717  67.752417     0.498           55
2014-09-08 06:42:00  70.686517  67.561983     0.787           55
2014-09-08 07:14:00  70.685450  67.548317     0.337           55

                     Minimum_Distance  Average_Distance        Kp  \
DateTime
2014-09-08 05:54:00        327.950987        665.008368  1.308696
2014-09-08 06:10:00        340.038476        667.146029  1.308696
2014-09-08 06:26:00        348.223318        678.815409  1.190909
2014-09-08 06:42:00        355.472899        680.040733  1.190909
2014-09-08 07:14:00        355.980432        680.062802  1.190909
```

```
                              N            E             C            H  \
DateTime
2014-09-08 05:54:00  6949.105566  3851.158789  57703.400422  7944.903535
2014-09-08 06:10:00  6985.653894  3866.337781  57645.097979  7984.229967
2014-09-08 06:26:00  7035.551690  3877.443099  57609.988412  8033.277823
2014-09-08 06:42:00  7082.978312  3886.943680  57575.069804  8079.412908
2014-09-08 07:14:00  7086.091085  3888.485656  57584.964457  8082.883617

                            D          I             F  \
DateTime
2014-09-08 05:54:00  28.994996  82.160510  58247.780322
2014-09-08 06:10:00  28.963136  82.114324  58195.405739
2014-09-08 06:26:00  28.860100  82.061723  58167.381903
2014-09-08 06:42:00  28.756769  82.011941  58139.191393
2014-09-08 07:14:00  28.755736  82.009909  58149.472389

                                     geometry
DateTime
2014-09-08 05:54:00  POINT (68.30733 70.85472)
2014-09-08 06:10:00  POINT (67.97505 70.83030)
2014-09-08 06:26:00  POINT (67.75242 70.76172)
2014-09-08 06:42:00  POINT (67.56198 70.68652)
2014-09-08 07:14:00  POINT (67.54832 70.68545)
```

[17]:

```
world = geopandas.read_file(geopandas.datasets.get_path('naturalearth_lowres'))
ax = world.plot(color='white', edgecolor='black', figsize = (12,6))

minx, miny, maxx, maxy = gdf.total_bounds
ax.set_xlim(minx, maxx)
ax.set_ylim(miny, maxy)

# We can now plot our ``GeoDataFrame``.
gdf.plot(ax=ax, column='F', legend=True,
         legend_kwds={'label': "Magnetic Intensity in nT",
                      'orientation': "horizontal"})
plt.ylabel("Longitude", fontsize=9)
plt.xlabel("Latitude", fontsize=9)

plt.show()
```

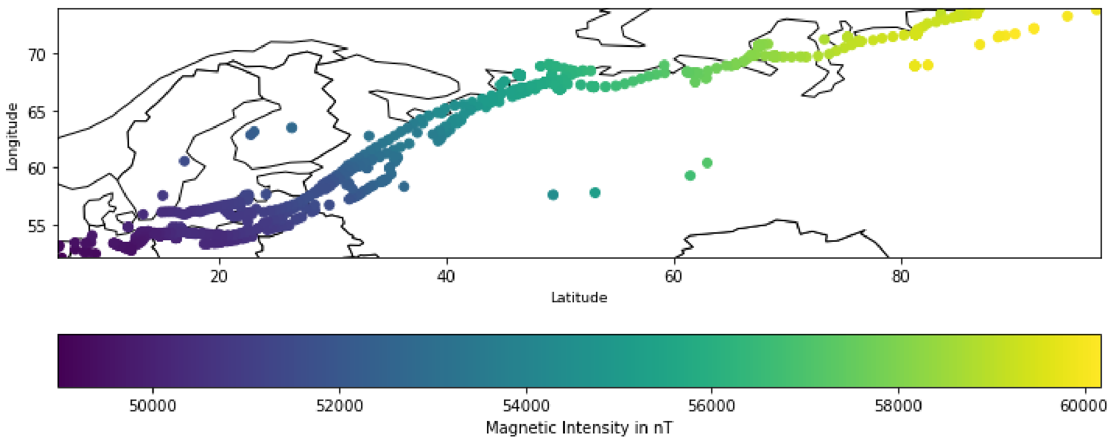

[18]:
```
fig, (ax1, ax2) = plt.subplots(ncols=2, figsize = (15,6))

ax1 = world.plot(ax=ax1, color='white', edgecolor='black')
xlim = ([gdf.total_bounds[0],  gdf.total_bounds[2]])
ylim = ([gdf.total_bounds[1],  gdf.total_bounds[3]])
ax1.set_xlim(xlim)
ax1.set_ylim(ylim)


gdf.plot(ax=ax1, column='F', legend=True,
         legend_kwds={'label': "Magnetic Intensity in nT",
                      'orientation': "horizontal"})
plt.ylabel("Longitude", fontsize=9)
plt.xlabel("Latitude", fontsize=9)
ax1.set_title('Magnetic Intensity - F')
ax1.set_xlabel('Latitude')
ax1.set_ylabel('Longitude')


ax2 = world.plot( ax=ax2, color='white', edgecolor='black')
xlim = ([gdf.total_bounds[0],  gdf.total_bounds[2]])
ylim = ([gdf.total_bounds[1],  gdf.total_bounds[3]])
ax2.set_xlim(xlim)
ax2.set_ylim(ylim)


# We can now plot our ``GeoDataFrame``.
gdf.plot(ax=ax2, column='D', legend=True, cmap='Spectral',
         legend_kwds={'label': " Declination in Degrees",
                      'orientation': "horizontal"})
ax2.set_title('Declination - D')
ax2.set_xlabel('Latitude')
```

```
ax2.set_ylabel('Longitude')
```

[18]: Text(567.7954545454544, 0.5, 'Longitude')

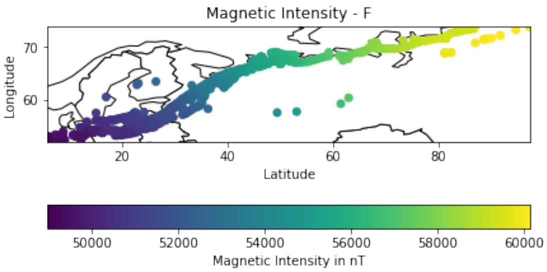

Supplement: Supplementary file 2 — Additional file 2. Code as Jupyter notebook (both sequential and parallel versions). [file 40462_2021_268_MOESM2_ESM.pdf]
